# Thermally Reentrant Crystalline Phase Change in Perovskite‐Derivative Nickelate Enabling Reversible Switching of Room‐Temperature Electrical Resistivity

**DOI:** 10.1002/advs.202304978

**Published:** 2023-09-03

**Authors:** Kota Matsumoto, Hideyuki Kawasoko, Eiji Nishibori, Tomoteru Fukumura

**Affiliations:** ^1^ Department of Chemistry Graduate School of Science Tohoku University Sendai 980‐8578 Japan; ^2^ PRESTO Japan Science and Technology Agency Saitama 332‐0012 Japan; ^3^ Department of Physics and Tsukuba Research Center for Energy Materials Science Faculty of Pure and Applied Sciences University of Tsukuba Tsukuba 305‐8571 Japan; ^4^ Advanced Institute for Materials Research and Core Research Cluster Tohoku University Sendai 980‐8577 Japan; ^5^ Center for Science and Innovation in Spintronics Tohoku University Sendai 980‐8577 Japan

**Keywords:** crystalline phase change, electrical conduction, local atomic arrangement, reversible switching of electrical resistivity, transition metal oxide

## Abstract

Reversible switching of room‐temperature electrical resistivity due to crystal‐amorphous transition is demonstrated in various chalcogenides for development of non‐volatile phase change memory. However, such reversible thermal switching of room‐temperature electrical resistivity has not reported in transition metal oxides so far, despite their enormous studies on the electrical conduction like metal‐insulator transition and colossal magnetoresistance effect. In this study, a thermally reversible switching of room‐temperature electrical resistivity is reported with gigantic variation in a layered nickelate Sr_2.5_Bi_0.5_NiO_5_ (1201‐SBNO) composed of (Sr_1.5_Bi_0.5_)O_2_ rock‐salt and SrNiO_3_ perovskite layers via unique crystalline phase changes between the conducting 1201‐SBNO with ordered (O‐1201), disordered Sr/Bi arrangements in the (Sr_1.5_Bi_0.5_)O_2_ layer (D‐1201), and insulating oxygen‐deficient double perovskite Sr_2_BiNiO_4.5_ (d‐perovskite). The O‐1201 is reentrant by high‐temperature annealing of ≈1000 °C through crystalline phase change into the D‐1201 and d‐perovskite, resulting in the thermally reversible switching of room‐temperature electrical resistivity with 10^2^‐ and 10^9^‐fold variation, respectively. The 1201‐SBNO is the first oxide to show the thermally reversible switching of room‐temperature electrical resistivity via the crystalline phase changes, providing a new perspective on the electrical conduction for transition metal oxides.

## Graphical abstract

A layered nickelate Sr_2.5_Bi_0.5_NiO_5_ is composed of rock‐salt (Sr_1.5_Bi_0.5_)O_2_ and perovskite SrNiO_3_ layers. This compound shows unique reentrant crystalline phase changes between ordered‐disordered Sr/Bi arrangements in the rock‐salt (Sr_1.5_Bi_0.5_)O_2_ layer and double perovskite Sr_2_BiNiO_4.5_ by air‐annealing. These phase changes enable thermally reversible switching of electrical conduction as is hardly shown in transition metal oxides.

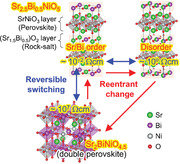

## 1 Introduction

Reversible switching of room‐temperature electrical resistivity due to crystal‐amorphous transition has been extensively studied in various chalcogenides such as Ge_2_Sb_2_Te_5_ for development of non‐volatile phase change memory.^[^ ^1^, ^2^, ^3^, ^4^, ^5^, ^6^, ^7^ ^]^ In transition metal oxides, large variation of electrical conduction has been extensively studied,^[^ ^8^ ^]^ as exemplified by metal‐insulator transition in perovskite‐type (La_1−_ *_x_* Sr*_x_*)TiO_3_ and *RE*NiO_3_ (*RE* = rare earth) via the control of band filling and width, and in VO_2_ and Magnéli phase *M_n_* O_2_ *_n_*_−1_ (*M* = Ti and V) via the structural phase transition.^[^ ^9^, ^10^, ^11^, ^12^ ^]^ The application of external stimuli such as magnetic field has also enabled the large variation of electrical conduction in transition metal oxides such as colossal magnetoresistance effect in La_0.7_Sr_0.3_MnO_3_.^[^ ^13^, ^14^ ^]^ In other transition metal oxides like double perovskite PrBaMn_2_O_6_ and La_2_CoMnO_6_,^[^ ^15^, ^16^, ^17^, ^18^ ^]^ local cation arrangements have been controlled by using high‐pressure synthesis to vary their electrical conduction. One noteworthy example is a 1201‐type layered (Cu_0.5_Cr_0.5_)Sr_2_CuO_5_ composed of alternate stack of rock‐salt (Cu_0.5_Cr_0.5_)SrO_2_ and perovskite SrCuO_3_ layers, where the ordered and disordered Cu/Cr arrangement in the rock‐salt layer selectively formed according to the cooling process in the high‐pressure synthesis, resulting in fivefold variation in electrical resistivity near room temperature.^[^ ^19^ ^]^ However, the reversible thermal switching of room‐temperature electrical resistivity, which was reported in various chalcogenides, has not been achieved in transition metal oxides so far.

A 1201‐type Sr_2.5_Bi_0.5_NiO_5_ (1201‐SBNO), composed of an alternate stack of rock‐salt (Sr_1.5_Bi_0.5_)O_2_ with ordered Sr/Bi arrangement and perovskite SrNiO_3_ layers, was reported to show low electrical resistivity of 2 × 10^−2^ Ωcm at room temperature,^[^ ^20^ ^]^ while most of other 1201‐type transition metal oxides such as Fe and Co oxides with ordered cation arrangement in rock‐salt layer exhibit insulating behavior.^[^ ^21^, ^22^, ^23^, ^24^ ^]^ Interestingly, a large thermal hysteresis of electrical resistivity was reported for only 1201‐SBNO, possibly suggesting thermal changes of the Sr/Bi arrangement and/or crystalline phase. In this study, we found unique crystalline phase changes in the 1201‐SBNO by annealing: 1201‐SBNO showed systematic change of the Sr/Bi arrangements from ordered to disordered states by the low‐temperature annealing up to 600 °C, underwent a crystalline phase change into novel oxygen‐deficient double perovskite Sr_2_BiNiO_4.5_ by the intermediate‐temperature annealing of 700–900 °C, and returned to the original 1201‐SBNO with the ordered Sr/Bi arrangement at high‐temperature annealing of 950–1100 °C. The ordered and disordered Sr/Bi arrangements in the 1201‐SBNO were changed by the annealing of 600 and 950 °C, respectively, resulting in a thermally reversible switching of room‐temperature electrical resistivity with 10^2^‐fold variation. Such thermally reversible switching of room‐temperature electrical resistivity was enhanced to be 10^9^‐fold variation via the crystalline phase change between the conducting 1201‐SBNO and insulating double perovskite Sr_2_BiNiO_4.5_ by the temperature‐programed annealing of 800 and 950 °C, respectively.

## 2 Results and Discussion

**Figure 1:**
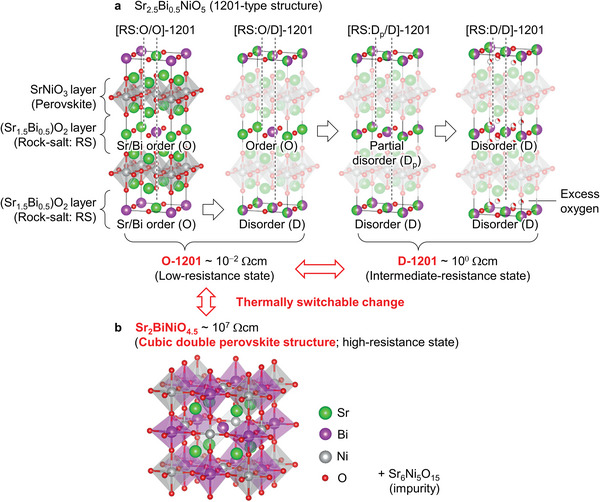
a) Crystal structure and corresponding electrical resistivity at room temperature for 1201‐SBNO depending on Sr/Bi arrangements and b) those for d‐perovskite. [RS:D/D]−1201 includes excess oxygen at 16 m site. O‐1201 denotes the mixed phase of [RS:O/O]‐ and [RS:O/D]−1201. D‐1201 denotes the mixed phase of [RS:D_p_/D]‐ and [RS:D/D]−1201.

**Figure** **1** shows schematic crystal structures of 1201‐SBNO controlled by air‐annealing: four‐type Sr/Bi arrangements of rock‐salt (Sr_1.5_Bi_0.5_)O_2_ layer in 1201‐SBNO (Figure 1a) and d‐perovskite (Figure 1b), where Rietveld analysis was used to determine these crystal structures including the crystallographic sites (Figure S1, Supporting Information). In Figure 1a, [RS:O/O]−1201 denotes the fully ordered 1201‐SBNO with ordered Sr/Bi arrangements at both *z* = 0 and 0.5, and [RS:O/D]−1201 denotes the partially ordered 1201‐SBNO with disordered and ordered Sr/Bi arrangements at *z* = 0 and 0.5, respectively. Also, [RS:D_p_/D]−1201 denotes the partially disordered 1201‐SBNO with disordered and partially disordered Sr/Bi arrangements at *z* = 0 and 0.5, respectively, and [RS:D/D]−1201 denotes the fully disordered 1201‐SBNO with disordered Sr/Bi arrangements at both *z* = 0 and 0.5, containing excess oxygen located between rock‐salt and perovskite layers. In Figure 1b, the crystal structure of novel oxygen‐deficient d‐perovskite phase consisted of Sr at 8 g site and ordered Sr/Bi arrangements at 1a/1b/3c/3d sites, in which octahedral oxygen coordinated with body centered Bi were deficient. As described below, [RS:O/O]‐ and [RS:O/D]‐ 1201 mixed phase, denoted as O‐1201, was low‐resistance state (≈10^−2^ Ωcm at 300 K). On the other hand, [RS:D_p_/D]‐ and [RS:D/D]‐ 1201 mixed phase, denoted as D‐1201, was intermediate‐resistance state (≈10^0^ Ωcm at 300 K). Furthermore, d‐perovskite was a high‐resistance state (≈10^7^ Ωcm at 300 K). Their room‐temperature electrical resistivity was reversibly switched via the thermal change of Sr/Bi arrangements or crystalline phases.

**Figure 2:**
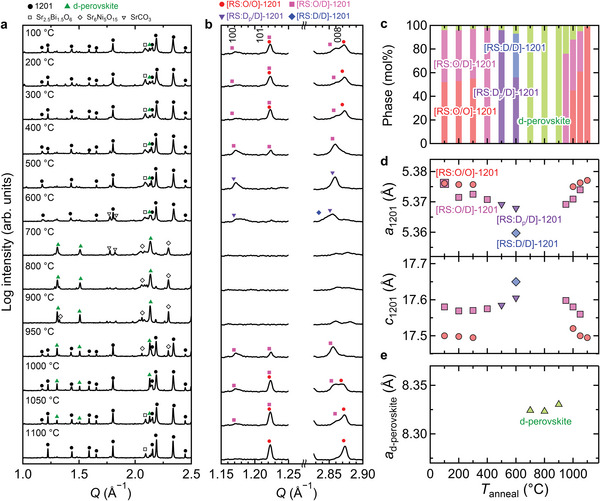
a) Synchrotron XRD patterns for different *T*_anneal_ between 100–1100 °C, and b) their magnified XRD patterns ≈100, 101, and 008 diffraction peaks. c) Molar fraction of 1201‐SBNO and d‐perovskite phases calculated by Rietveld analysis, d) *a*‐ and *c*‐axis lengths for 1201‐SBNO, and e) *a*‐axis length for d‐perovskite as a function of *T*_anneal_.

**Figure 3:**
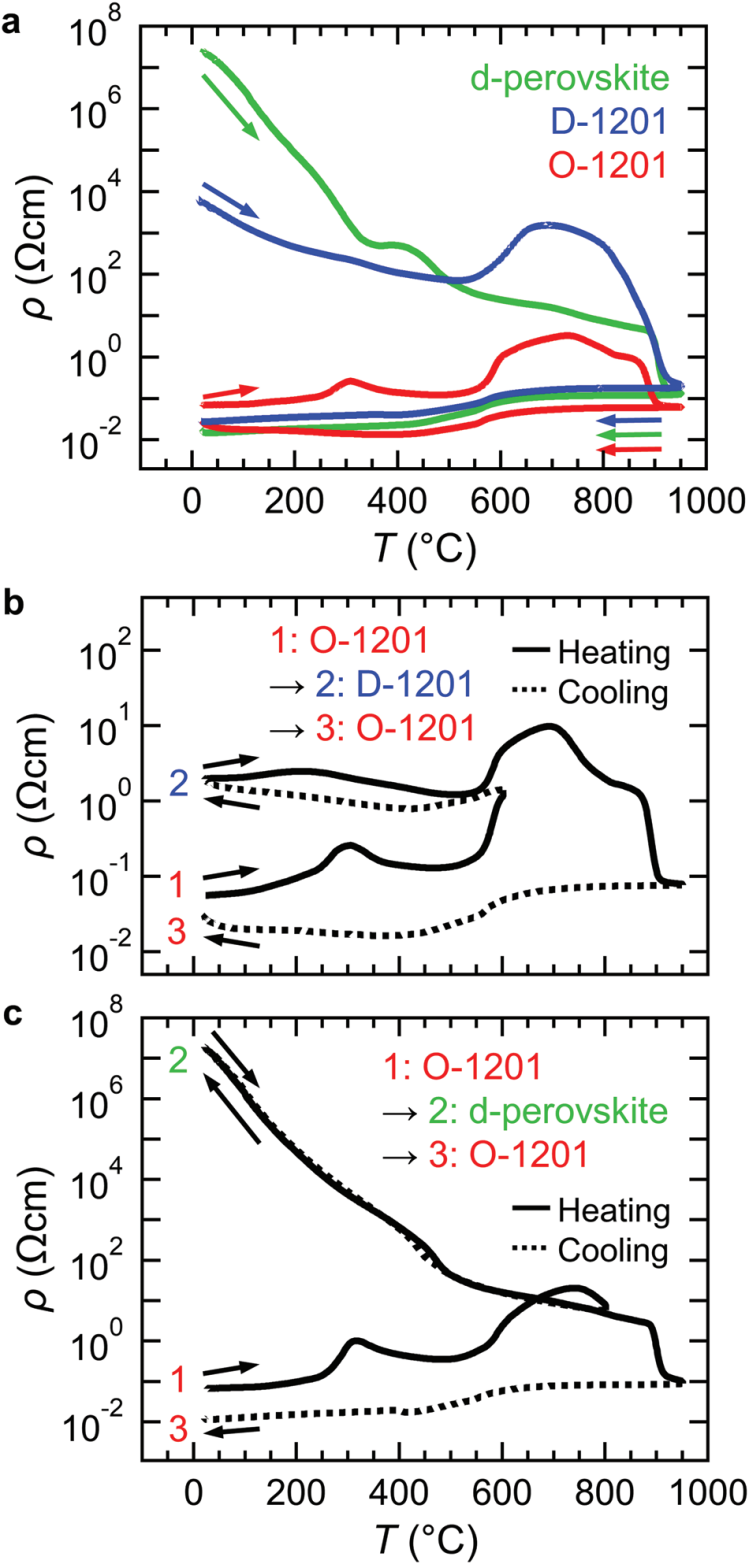
a) Temperature dependence of electrical resistivity above room temperature for O‐1201, D‐1201, and d‐perovskite. Temperature dependence of electrical resistivity during crystalline phase change b) between O‐ and D‐1201 and c) between O‐1201 and d‐perovskite. The numbers in each panel denote heating/cooling steps, where corresponding XRD patterns are shown in Figure S7 (Supporting Information). Solid and dotted lines correspond to heating and cooling processes, respectively.

**Figure** **2**a shows the synchrotron X‐ray diffraction (XRD) patterns of O‐1201 after annealing for *T*_anneal_ = 100–1100 °C (see Figures S2–S4, and Tables S1–S4, Supporting Information, for the detailed Rietveld analysis). Most of diffraction peaks for *T*_anneal_ = 100–600 °C were attributed to the crystal structures of 1201‐SBNO, while those for 700–900 °C were attributed to d‐perovskite, indicating the crystalline phase change at ≈ 700°C. For *T*_anneal_ ≥ 950 °C, the diffraction peaks of the 1201‐SBNO appeared again in place of those of d‐perovskite, representing a reentrant crystalline phase change for 1201‐SBNO. The crystalline phase change in the 1201‐SBNO and d‐perovskite phases was accompanied with the formation of tiny impurity phase of Sr_6_Ni_5_O_15_ as seen in the small diffraction peaks. However, the Sr_6_Ni_5_O_15_ would have the negligible influence on the electrical conduction in **Figure** **3**.

The Sr/Bi arrangements in the (Sr_1.5_Bi_0.5_)O_2_ rock‐salt layer in 1201‐SBNO possessed ordered and/or disordered states as a function of *T*_anneal_ as follows. Figure 2b shown the magnified XRD patterns ≈100, 101, and 008 diffraction peaks for 1201‐SBNO as indicators of the Sr/Bi arrangements: 100 peak denotes the presence of either [RS:O/D]‐ or [RS:D_p_/D]−1201 phase due to the space group of P4/*mmm*, 101 peak intensity is proportional to the degree of ordering for Sr/Bi arrangements, and split 008 peaks denote mixed phase of 1201‐SBNO with different Sr/Bi arrangements. The strongest 101 peak intensity without 100 peak corresponds to [RS:O/O]−1201 with the space group of *I*4/*mmm*; on the other hand, neither 101 nor 100 peak corresponds to [RS:D/D]−1201 with the space group of *I*4/*mmm*. For *T*_anneal_ = 100–300 °C, the 100 and 101 peaks indicated the presence of both [RS:O/D]‐ and [RS:O/O]−1201, consistent with the split 008 peaks at *Q* = 2.85 and 2.87 Å^−1^, respectively. From this result, the Sr/Bi arrangements were not changed for *T*_anneal_ ≤ 300 °C. For *T*_anneal_ = 400 °C, the single 008 peak at *Q* = 2.85 Å^−1^ with smaller 101 peak indicates the presence of only [RS:O/D]−1201 as a result of the disappearance of [RS:O/O]−1201. For *T*_anneal_ = 500 °C, disappearance of the 101 peak indicates partially disordered state of the Sr/Bi arrangements at *z* = 0.5 ([RS:D_p_/D]−1201; Figure 1). For *T*_anneal_ = 600 °C, the split 008 peaks at *Q* = 2.83 and 2.85 Å^−1^ with smaller 100 peak indicates the presence of D_f_‐ and D_p_‐1201, corresponding to D‐1201. For *T*_anneal_ = 950 °C, only the [RS:O/D]−1201 was formed, and for *T*_anneal_ ≥ 950 °C, the [RS:D_p_/D]‐ and [RS:D/D]−1201 were absent. For the higher *T*_anneal_, the split 008 peaks at *Q* = 2.85 and 2.87 Å^−1^ with the smaller 100 peak indicates the presence of not only [RS:O/D]‐ but also [RS:O/O]−1201. For *T*_anneal_ = 1100°C, the single 008 peak at *Q* = 2.87 Å^−1^ without 100 peak indicates the presence of only [RS:O/O]−1201. To summarize, the Sr/Bi arrangements in 1201‐SBNO changed from ordered to disordered state for *T*_anneal_ ≤ 600 °C, but returned to be ordered state for *T*_anneal_ ≥950 °C, through intermediate temperature phase, d‐perovskite for 700°C ≤ *T*_anneal_ ≤ 900°C. These phase fraction calculated from Rietveld analysis is shown in Figure 2c and Table S4 (Supporting Information). It is noted that these crystalline phase changes are distinct from the well‐known structural change between perovskite and brownmillerite caused by oxygen non‐stoichiometry.^[^ ^25^ ^]^

Figure 2d,e shows the lattice constants of 1201‐SBNO and d‐perovskite with different *T*_anneal_. For each phase including d‐perovskite phase, the *a*‐ and *c*‐axis lengths were almost constant irrespective of *T*_anneal_, and the disordering of the Sr/Bi arrangements in 1201‐SBNO resulted in shorter *a*‐axis length and longer *c*‐axis length. Taking into account almost stoichiometric molar ratio of Sr, Bi, and Ni for 1201‐SBNO with all *T*_anneal_ (Table S5, Supporting Information; Sr:Bi:Ni = 2.5:0.5:1.0) evaluated by the energy dispersive X‐ray spectroscopy (SEM‐EDX), this change in the lattice constants was mainly attributed to the different Sr/Bi arrangements (Figure S5, Supporting Information). In addition, the smallest *a*‐ and largest *c*‐axis lengths for [RS:D/D]−1201 were partially caused by the excess oxygen.

From the thermogravimetric (TG) analysis for O‐1201 in air, the weight once decreased during heating, and then almost returned to the original value after cooling (Figure S6, Supporting Information), indicating the vaporization of cations was unlikely to occur during annealing. This result is consistent with the unchanged cationic composition before and after annealing, confirmed by SEM‐EDX results (Table S5, Supporting Information). The weight change was probably caused by variable oxygen stoichiometry via redox reaction with oxygen in air during annealing. Indeed, O‐1201 with trivalent Ni ion underwent a crystalline phase change into the d‐perovskite with divalent Ni ion by annealing at 700–900 °C. The Ni ion was further reduced by heating above 900 °C, and was oxidized during cooling down to at room temperature, probably resulting in the reentrant formation of the O‐1201. Also, we confirmed that the crystalline structure was repeatedly restored under 12 cycles of heating and cooling of between room temperature and 1000 °C by performing additional XRD experiments (Figure S7 and Tables S6 and S7, Supporting Information).

Figure 3a shows the temperature dependence of the electrical resistivity during heating and cooling above room temperature for O‐1201, D‐1201, and d‐perovskite, where the room‐temperature electrical resistivity before heating was 6.9 × 10^−2^, 6.5 × 10^3^, and 2.5 × 10^7^ Ωcm, respectively. For the O‐1201, the electrical resistivity during heating was rather temperature independent up to 550 °C except for a small kink at 300 °C, turned to increase up to 750 °C and decrease above 750 °C, and steeply decreased above 900 °C. For the D‐1201, the electrical resistivity during heating monotonically decreased up to 550 °C, and showed similar temperature dependence above 550 °C to that of the O‐1201 despite the large difference in their electrical resistivity. The [RS:O/O]−1201 formed by annealing at 1100 °C was too brittle for its electrical resistivity measurement. However, similar room‐temperature electrical resistivity between the [RS:O/O]−1201 (2 × 10^−2^ Ωcm;^[20^ ^]^) and the O‐1201 (6.9 × 10^−2^ Ωcm; Figure 3a) indicates the insignificant difference in electrical resistivity between these two phases. For the d‐perovskite, the room‐temperature electrical resistivity was much higher than that for O‐ and D‐1201, indicating the insulating ground state. Also, the monotonical decrease of the electrical resistivity during heating up to 900 °C was reasonably explained by the thermal excitation of conduction carriers in the insulator. Since most of divalent nickelates such as NiO are insulators,^[^ ^26^ ^]^ the d‐perovskite with divalent Ni could be also one of the examples. Above 900 °C, the electrical resistivity of the d‐perovskite steeply decreased above 900 °C like the O‐ and D‐1201. All these phases showed the similar resistivity during cooling below 950 °C with metallic temperature dependence, caused by their phase change into O‐ or [RS:O/O]−1201 according to XRD analysis (Figure S6 and Table S6, Supporting Information), being consistent with the SXRD analysis in Figure 2. Also, the 1201‐SBNO was paramagnet irrespective of the Sr/Bi arrangements in (Sr_1.5_Bi_0.5_)O_2_ rock‐salt layer, while the d‐perovskite was a canted antiferromagnet with a transition temperature of 20 K according to the magnetization measurements (Figure S8, Supporting Information).

Here, we show the thermally reversible switching of room‐temperature electrical resistivity with gigantic variation via the crystalline phase changes between O‐1201, D‐1201, and d‐perovskite. Figure 3b shows the temperature dependence of the electrical resistivity in the phase change between O‐ and D‐1201. In this phase change, the initial phase of O‐1201 (Figure S7a and Table S7a, Supporting Information) was heated up to 600 °C, and cooled down to room temperature. Subsequently, it was heated up to 950 °C, and cooled down to room temperature. After the first heating and cooling, the room‐temperature electrical resistivity increased to be 1.7 × 10^0^ Ωcm which was 10^2^‐fold higher than that of O‐1201, indicating the phase change into D‐1201 (Figure S7b and Table S7b, Supporting Information). After the second heating and cooling, the room‐temperature electrical resistivity decreased to be 2.5 × 10^−2^ Ωcm, corresponding to the phase change into O‐1201 (Figure S7c and Table S7c, Supporting Information).

Figure 3c shows the temperature dependence of the electrical resistivity in the crystalline phase change between O‐1201 and d‐perovskite. In this phase change, the initial phase of O‐1201 (Figure S7a and Table S7a, Supporting Information) was heated up to 800 °C, and cooled down to room temperature. Subsequently, it was heated up to 950 °C, and cooled down to room temperature. After the first heating and cooling, the room‐temperature electrical resistivity increased to be 1.9 × 10^7^ Ωcm, which is 10^9^‐fold higher than that of O‐1201. This was attributed to the phase change into d‐perovskite, confirmed by XRD measurement (Figure S7d and Table S7d, Supporting Information). In the second heating and cooling, the temperature dependence of the electrical resistivity was almost similar to that of d‐perovskite as shown in Figure 3a, indicating the phase change into O‐1201 (Figure S7e and Table S7e, Supporting Information). Also, the reversible switching of room‐temperature electrical resistivity in 1201‐SBNO at lower temperatures would pave the way to applications for nonvolatile phase change as demonstrated in in chalcogenides.

**Figure 4:**
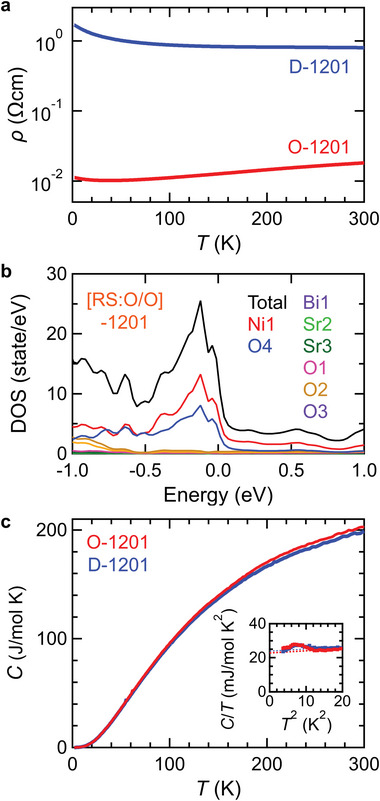
a) Temperature dependence of electrical resistivity below room temperature for O‐ and D‐1201. b) total/partial density of states for [RS:O/O]−1201. c) Temperature dependence of specific heat for O‐ and D‐1201. Inset shows *C*/*T* versus *T* ^2^ plot; the anomaly at around *T* ^2^ = 9 K^2^ was probably caused by a phase transition of an impurity phase because of no anomaly of the electrical resistivity and magnetic susceptibility at around *T* = 3 K.

Finally, we discuss the origin of the large variation in the electrical resistivity for the O‐ and D‐1201 with the physical properties below 300 K in Figures 4a–c. For the O‐1201, the electrical resistivity decreased with decreasing temperature, indicating the metallic conduction (**Figure** **4**a). For the D‐1201, on the other hand, the electrical resistivity monotonically increased with decreasing temperature (Figure 4a), suggesting the insulator‐like behavior. The O‐1201 had the 10^2^‐fold lower electrical resistivity than the D‐1201, being consistent with Figure 3c. The band structure of [RS:O/O]−1201 has a metallic ground state possessing several band crossings at the Fermi energy (Figure S8, Supporting Information), that are mainly composed of the Ni1 and O4 orbitals for NiO_2_ plane of SrNiO_3_ perovskite layer in [RS:O/O]−1201 (Figure 4b), suggesting electrically conducting SrNiO_3_ perovskite layer and insulating (Sr_1.5_Bi_0.5_)O_2_ rock‐salt layer. For the O‐1201, the specific heat (*C*) exhibited the featureless temperature dependence without phase transition below 300 K (Figure 4c). Also, the electronic specific heat coefficient of 23 mJ mol^−1^ K^−2^ obtained from *C*/*T* versus *T* ^2^ plot (inset of Figure 4c; Table S8, Supporting Information) was almost consistent with that of 30 mJ mol^−1^ K^−2^ calculated by the density of states at Fermi energy (Figure 4b) on the assumption of free electron model, indicating the metallic ground state. It is noted that the specific heat coefficient was comparable to other metallic nickelates such as LaNiO_3_.^[^ ^27^, ^28^, ^29^, ^30^ ^]^ Despite the higher electrical resistivity with insulator‐like behavior, the specific heat of the D‐1201 was quite similar to that of the O‐1201; the featureless temperature dependence indicated no phase transition below 300 K (Figure 4c), and the electronic specific heat coefficient of 24 mJ mol^−1^ K^−2^ represented the metallic ground state (inset of Figure 4c; Table S6, Supporting Information), suggesting the similar carrier density of O‐ and D‐1201. Accordingly, these physical properties below 300 K indicate that 1201‐SBNO is a nonmagnetic metal irrespective of the Sr/Bi arrangements in (Sr_1.5_Bi_0.5_)O_2_ rock‐salt layer, supported by the magnetic susceptibility (Figure S5, Supporting Information). Despite of the metallic state for the O‐ and D‐1201, the D‐1201 showed the much higher electrical resistivity than the O‐1201 even at room temperature, suggesting that the high electrical resistivity was attributed to the randomness effect via the disordered Sr/Bi arrangements, known as resistivity saturation.^[^ ^31^ ^]^ Also, taking into account increased electrical resistivity and its similar temperature dependence in LaNiO_3_ ultrathin films,^[^ ^32^, ^33^ ^]^ the high electrical resistivity of the D‐1201 would originate from Anderson localization and/or the modified band structure due to the enhanced two‐dimensionality, which is caused by the weakened interlayer coupling between electrically conducting SrNiO_3_ perovskite layers via the disordered Sr/Bi arrangements. It is noted that the electrical resistivity below room temperature for the d‐perovskite was beyond measurement.

## 3 Conclusion

In summary, 1201‐SBNO was found to undergo crystalline phase changes in 1201‐SBNO with the different Sr/Bi arrangements and d‐perovskite by the low‐ and intermediate‐ temperature annealing, respectively, and to return to the original 1201‐SBNO at the high‐temperature annealing. Furthermore, the two kinds of thermally reversible switching of the room‐temperature electrical resistivity were realized with (1) 10^2^‐fold variation via the change between the ordered and disordered Sr/Bi arrangements in the 1201‐SBNO and (2) 10^9^‐fold variation via the crystalline phase change between the conducting 1201‐SBNO and insulating d‐perovskite. The 1201‐SBNO is the first oxide to show the thermally reversible switching of the room‐temperature electrical resistivity via the crystalline phase changes, providing a new perspective in the variation of electrical conduction for transition metal oxides and suggesting the potential application for oxide‐based phase change memory.

## 4 Experimental Section

All polycrystalline samples were synthesized by solid‐state reaction in air. SrO (98%; Kojundo Chemical), Bi_2_O_3_ (99.999%; Kojundo Chemical), and NiO (99.97%; Kojundo Chemical) powders were mixed and pelletized under 20 MPa to form nominal composition of Sr_2.5_Bi_0.5_NiO_4.25_ in nitrogen filled glove box. The pellets were sintered at 750 °C for 5 h, 900 °C for 15 h, and 1100 °C for 48 h, to form 1201‐SBNO by sintering in air, and cooled down to room temperature; the heating and cooling rate was fixed to be 200 °C h^−1^ in all the processes. The 1201‐SBNO was ground and pelletized under 30 MPa, followed by annealing in air at various temperature (100–1100 °C) for 24 h with heating and cooling rates of 200 °C h^−1^ to induce the crystal phase change. The crystal structures were evaluated at room temperature by XRD with synchrotron radiation (SPring‐8 BL02B2, wavelength: 0.52 Å) and Cu Kα radiation (D8 Discover, Bruker AXS; SmartLab, Rigaku). The crystal structural parameters were obtained by Rietveld analysis with RIETAN‐FP.^[^ ^34^ ^]^ The crystal structures were drawn with the VESTA.^[^ ^35^ ^]^ The chemical composition was evaluated by scanning electron microscopy equipped with energy dispersive X‐ray spectroscopy (SEM‐EDX; S‐4300 and TM4000, Hitachi). The electrical resistivity above room temperature was measured in air at a lamp furnace (MILA5000, Advance Riko, Inc.) with a source‐measure unit (Keithley 2460); the samples were hold at the set temperature for 10 min, with the heating and cooling rate of 180 °C h^−1^. The electrical resistivity, specific heat, and magnetic susceptibility below 300 K were measured by physical property measurement system (PPMS, Quantum Design) and magnetic property measurement system (MPMS, Quantum Design). The electronic structure was calculated by full potential linearized augmented plane wave method, implemented in the WIEN2k code.^[^ ^36^ ^]^

## Conflict of Interest

The authors declare no conflict of interest.

## Supporting information

Supporting Information

## Data Availability

The data that support the findings of this study are available from the corresponding author upon reasonable request.
