# Supplementary material for: Thermally Reentrant Crystalline Phase Change in Perovskite‐Derivative Nickelate Enabling Reversible Switching of Room‐Temperature Electrical Resistivity
Source: Adv Sci (Weinh). 2023 Sep 3;10(31):2304978. doi: 10.1002/advs.202304978 (PMC10625122; doi:10.1002/advs.202304978)
Supplement: Supplementary file 1 — Supporting Information [file ADVS-10-2304978-s001.pdf]

ADVANCED SCIENCE
Open Access

Supporting Information

for *Adv. Sci.*, DOI 10.1002/advs.202304978

Thermally Reentrant Crystalline Phase Change in Perovskite-Derivative Nickelate Enabling Reversible Switching of Room-Temperature Electrical Resistivity

Kota Matsumoto, Hideyuki Kawasoko, Eiji Nishibori and Tomoteru Fukumura**

Supporting information

Thermally reentrant crystalline phase change in perovskite-derivative nickelate enabling reversible switching of room-temperature electrical resistivity

Kota Matsumoto[1], Hideyuki Kawasoko[1,2,*], Eiji Nishibori[3], and Tomoteru Fukumura[1,4,5,*]

[1] Department of Chemistry, Graduate School of Science, Tohoku University, Sendai 980-8578, Japan

[2] PRESTO, Japan Science and Technology Agency, Saitama 332-0012, Japan

[3] Department of Physics and Tsukuba Research Center for Energy Materials Science, Faculty of Pure and Applied Sciences, University of Tsukuba, Tsukuba 305-8571, Japan.

[4] Advanced Institute for Materials Research and Core Research Cluster, Tohoku University, Sendai 980-8577, Japan

[5] Center for Science and Innovation in Spintronics, Tohoku University, Sendai 980-8577, Japan

* hideyuki.kawasoko.b7@tohoku.ac.jp

* tomoteru.fukumura.e4@tohoku.ac.jp

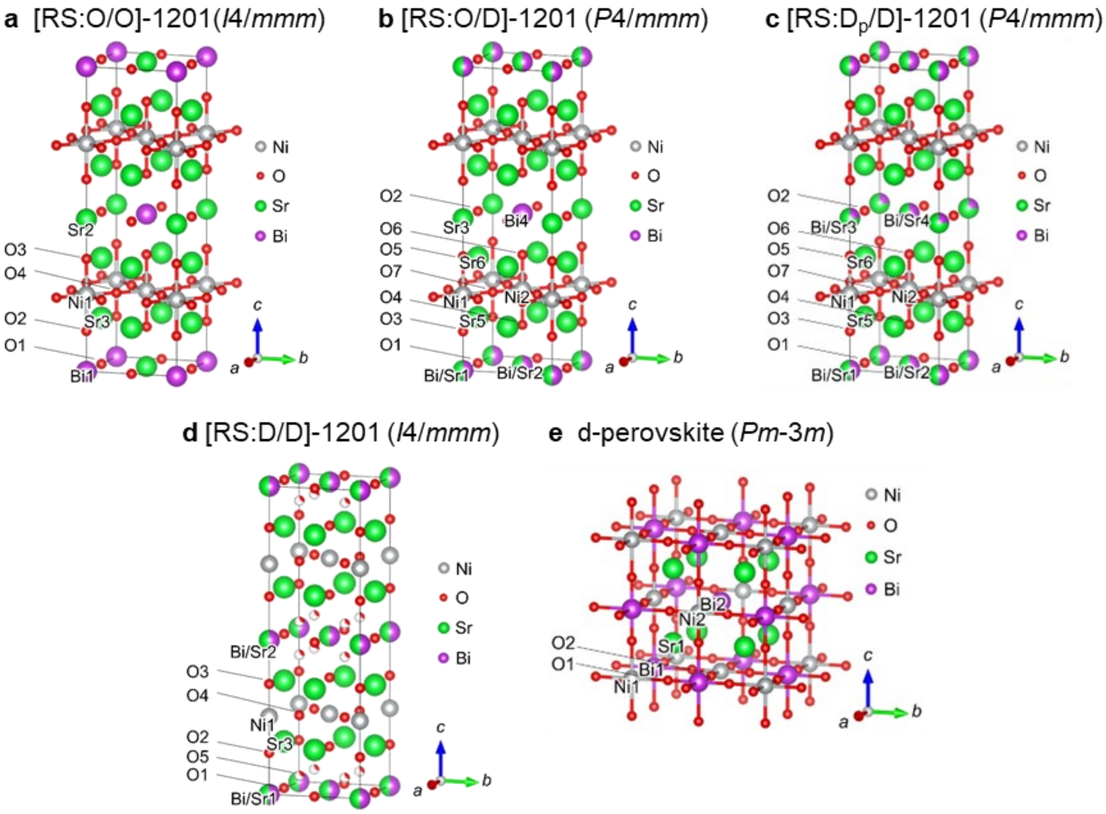

Figure S1. Crystal structure of a) [RS:O/O]-1201, b) [RS:O/D]-1201, c) [RS:D_p/D]-1201, d) [RS:D_p/D]-1201, and e) d-perovskite phases for SBNO. The atomic coordinates of Ni, O, Sr, and Bi in each crystal structure are listed in Tables S1, S2 and S3.

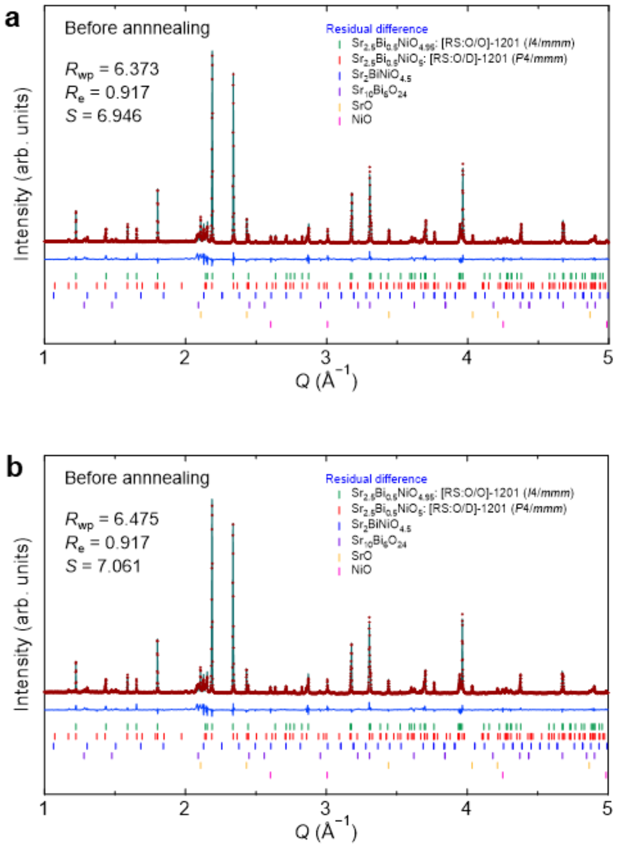

Figure S2. Synchrotron XRD patterns and the fitting results of Rietveld refinement for O-1201 before air-annealing. The X-ray wavelength was 0.78 Å. Brown and gray curves denote the measurement data and the simulation pattern. a) The Rietveld analysis was performed without any constraint. b) The occupancy of Sr/Bi was fixed as shown in Table S1b.

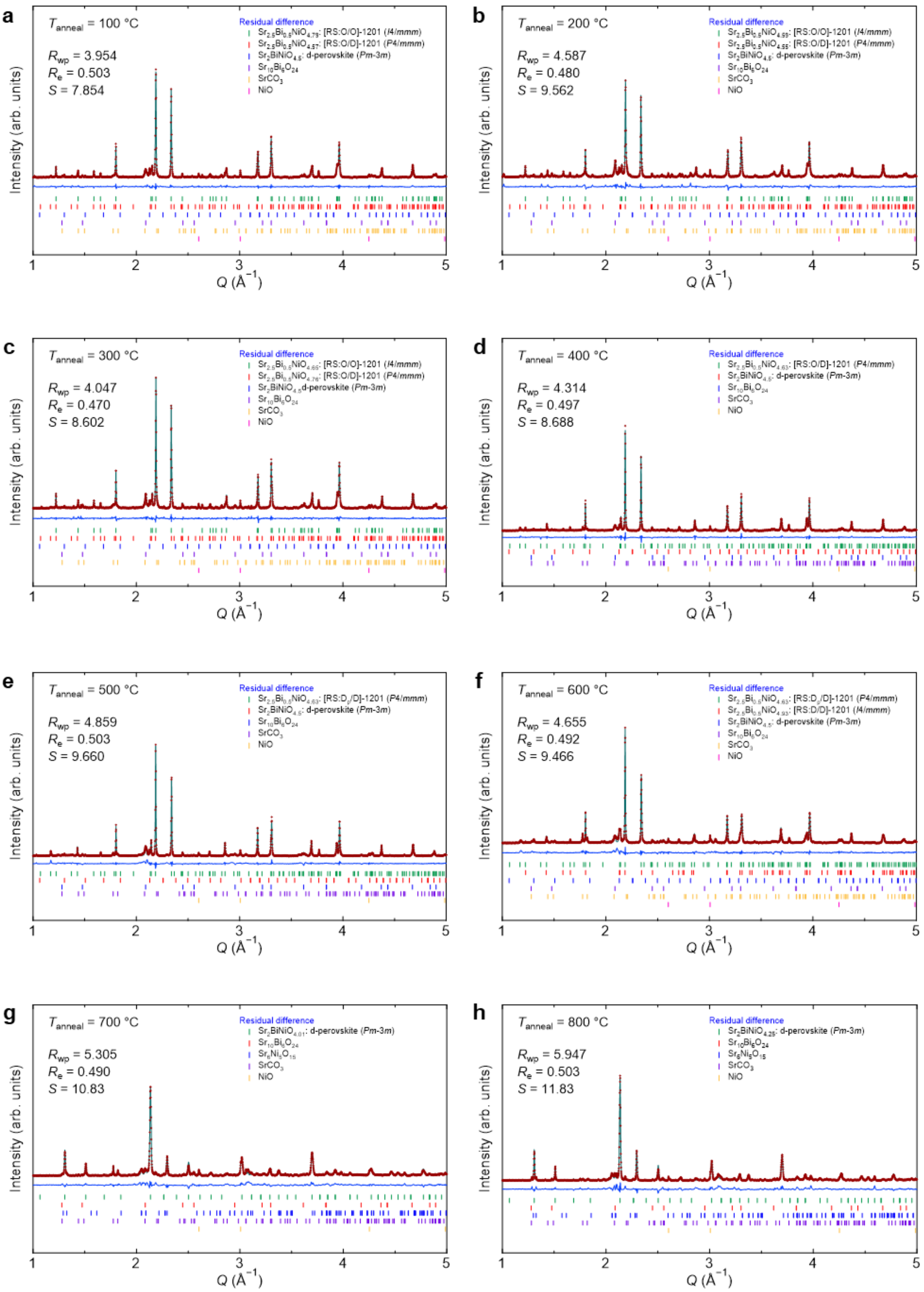

a
T_{anneal} = 100 °C
R_{wp} = 3.954
R_e = 0.503
S = 7.854
Residual difference
$Sr_{2.5}Bi_{0.5}NiO_{4.79}$: [RS:O/O]-1201 (I4/mmm)
$Sr_{2.5}Bi_{0.5}NiO_{4.57}$: [RS:O/D]-1201 (P4/mmm)
$Sr_2BiNiO_{4.5}$: d-perovskite (Pm-3m)
$Sr_{10}Bi_6O_{24}$
$SrCO_3$
NiO
b
T_{anneal} = 200 °C
R_{wp} = 4.587
R_e = 0.480
S = 9.562
Residual difference
$Sr_{2.5}Bi_{0.5}NiO_{4.59}$: [RS:O/O]-1201 (I4/mmm)
$Sr_{2.5}Bi_{0.5}NiO_{4.55}$: [RS:O/D]-1201 (P4/mmm)
$Sr_2BiNiO_{4.5}$: d-perovskite (Pm-3m)
$Sr_{10}Bi_6O_{24}$
$SrCO_3$
NiO
c
T_{anneal} = 300 °C
R_{wp} = 4.047
R_e = 0.470
S = 8.602
Residual difference
$Sr_{2.5}Bi_{0.5}NiO_{4.66}$: [RS:O/O]-1201 (I4/mmm)
$Sr_{2.5}Bi_{0.5}NiO_{4.76}$: [RS:O/D]-1201 (P4/mmm)
$Sr_2BiNiO_{4.5}$d-perovskite (Pm-3m)
$Sr_{10}Bi_6O_{24}$
$SrCO_3$
NiO
d
T_{anneal} = 400 °C
R_{wp} = 4.314
R_e = 0.497
S = 8.688
Residual difference
$Sr_{2.5}Bi_{0.5}NiO_{4.63}$: [RS:O/D]-1201 (P4/mmm)
$Sr_2BiNiO_{4.5}$: d-perovskite (Pm-3m)
$Sr_{10}Bi_6O_{24}$
$SrCO_3$
NiO
e
T_{anneal} = 500 °C
R_{wp} = 4.859
R_e = 0.503
S = 9.660
Residual difference
$Sr_{2.5}Bi_{0.5}NiO_{4.63}$: [RS:$D_p$/D]-1201 (P4/mmm)
$Sr_2BiNiO_{4.5}$: d-perovskite (Pm-3m)
$Sr_{10}Bi_6O_{24}$
$SrCO_3$
NiO
f
T_{anneal} = 600 °C
R_{wp} = 4.655
R_e = 0.492
S = 9.466
Residual difference
$Sr_{2.5}Bi_{0.5}NiO_{4.63}$: [RS:$D_p$/D]-1201 (P4/mmm)
$Sr_{2.5}Bi_{0.5}NiO_{4.93}$: [RS:D/D]-1201 (I4/mmm)
$Sr_2BiNiO_{4.5}$: d-perovskite (Pm-3m)
$Sr_{10}Bi_6O_{24}$
$SrCO_3$
NiO
g
T_{anneal} = 700 °C
R_{wp} = 5.305
R_e = 0.490
S = 10.83
Residual difference
$Sr_2BiNiO_{4.01}$: d-perovskite (Pm-3m)
$Sr_{10}Bi_6O_{24}$
$Sr_6Ni_5O_{15}$
$SrCO_3$
NiO
h
T_{anneal} = 800 °C
R_{wp} = 5.947
R_e = 0.503
S = 11.83
Residual difference
$Sr_2BiNiO_{4.25}$: d-perovskite (Pm-3m)
$Sr_{10}Bi_6O_{24}$
$Sr_6Ni_5O_{15}$
$SrCO_3$
NiO
Intensity (arb. units)
Q ($Å^{-1}$)
1
2
3
4
5

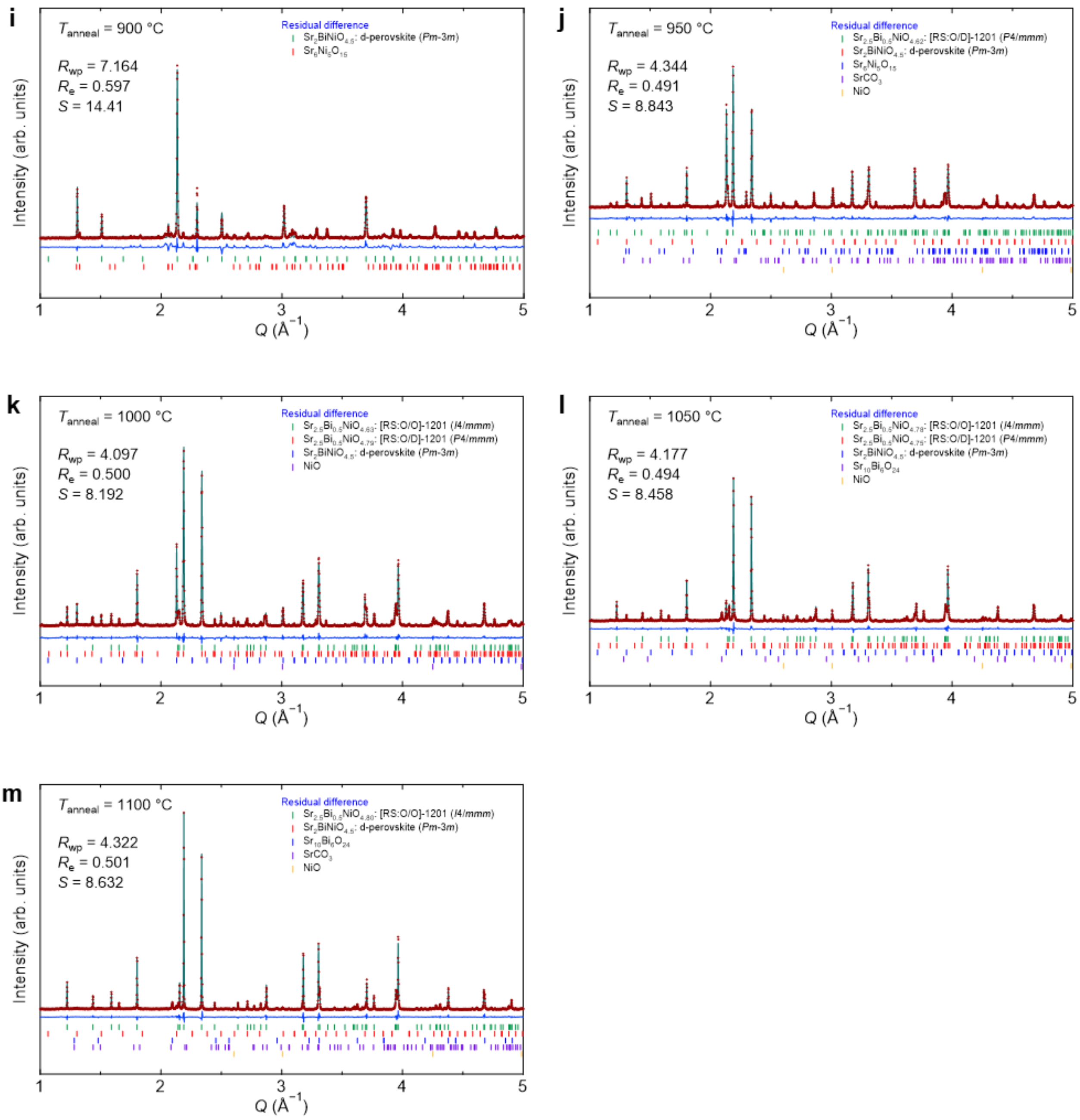

Figure S3. Synchrotron XRD patterns and the fitting results of Rietveld refinement for O-1201 with a) T_{anneal} = 100 °C, b) 200 °C, c) 300 °C, d) 400 °C, e) 500 °C, f) 600 °C, g) 700 °C, h) 800 °C, (i) 900 °C, (j) 950 °C, (k) 1000 °C, (l) 1050 °C, and (m) 1100 °C. Brown and gray curves denote the measurement data and the simulation pattern. The occupancy of Sr/Bi was fixed as shown in Table S2 by taking into account the continuity of temperature dependence. It is noted that these results are adopted in the main text.

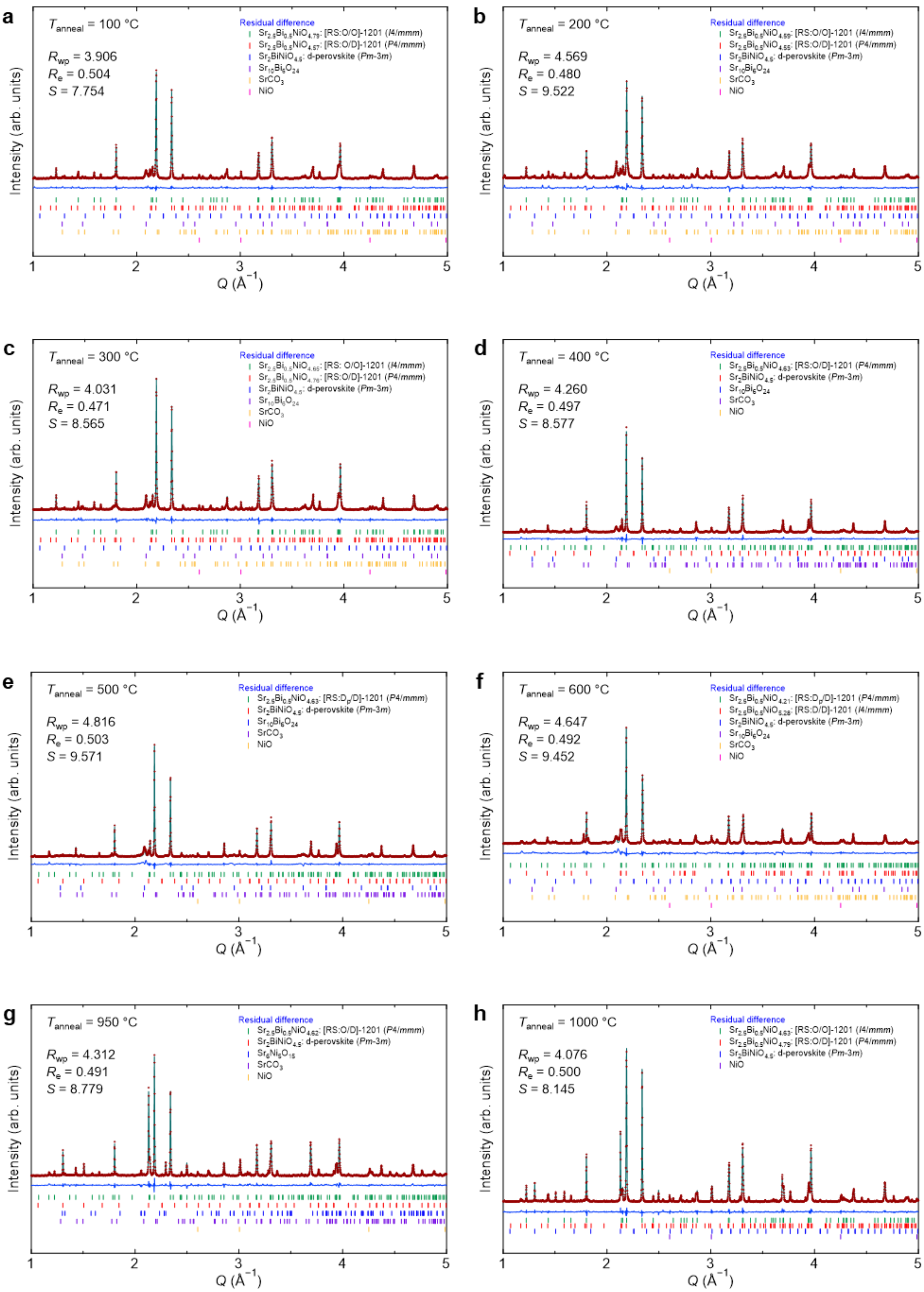
a
T_anneal = 100 °C
R_wp = 3.906
R_e = 0.504
S = 7.754
Residual difference
Sr2.5Bi0.5NiO4.79: [RS:O/O]-1201 (I4/mmm)
Sr2.5Bi0.5NiO4.57: [RS:O/D]-1201 (P4/mmm)
Sr2BiNiO4.5: d-perovskite (Pm-3m)
Sr10Bi6O24
SrCO3
NiO
b
T_anneal = 200 °C
R_wp = 4.569
R_e = 0.480
S = 9.522
Residual difference
Sr2.5Bi0.5NiO4.59: [RS:O/O]-1201 (I4/mmm)
Sr2.5Bi0.5NiO4.55: [RS:O/D]-1201 (P4/mmm)
Sr2BiNiO4.5: d-perovskite (Pm-3m)
Sr10Bi6O24
SrCO3
NiO
c
T_anneal = 300 °C
R_wp = 4.031
R_e = 0.471
S = 8.565
Residual difference
Sr2.5Bi0.5NiO4.65: [RS: O/O]-1201 (I4/mmm)
Sr2.5Bi0.5NiO4.76: [RS:O/D]-1201 (P4/mmm)
Sr2BiNiO4.5: d-perovskite (Pm-3m)
Sr10Bi6O24
SrCO3
NiO
d
T_anneal = 400 °C
R_wp = 4.260
R_e = 0.497
S = 8.577
Residual difference
Sr2.5Bi0.5NiO4.63: [RS:O/D]-1201 (P4/mmm)
Sr2BiNiO4.5: d-perovskite (Pm-3m)
Sr10Bi6O24
SrCO3
NiO
e
T_anneal = 500 °C
R_wp = 4.816
R_e = 0.503
S = 9.571
Residual difference
Sr2.5Bi0.5NiO4.63: [RS:Dp/D]-1201 (P4/mmm)
Sr2BiNiO4.5: d-perovskite (Pm-3m)
Sr10Bi6O24
SrCO3
NiO
f
T_anneal = 600 °C
R_wp = 4.647
R_e = 0.492
S = 9.452
Residual difference
Sr2.5Bi0.5NiO4.21: [RS:Dp/D]-1201 (P4/mmm)
Sr2.5Bi0.5NiO5.28: [RS:D/D]-1201 (I4/mmm)
Sr2BiNiO4.5: d-perovskite (Pm-3m)
Sr10Bi6O24
SrCO3
NiO
g
T_anneal = 950 °C
R_wp = 4.312
R_e = 0.491
S = 8.779
Residual difference
Sr2.5Bi0.5NiO4.62: [RS:O/D]-1201 (P4/mmm)
Sr2BiNiO4.5: d-perovskite (Pm-3m)
Sr6Ni5O15
SrCO3
NiO
h
T_anneal = 1000 °C
R_wp = 4.076
R_e = 0.500
S = 8.145
Residual difference
Sr2.5Bi0.5NiO4.63: [RS:O/O]-1201 (I4/mmm)
Sr2.5Bi0.5NiO4.79: [RS:O/D]-1201 (P4/mmm)
Sr2BiNiO4.5: d-perovskite (Pm-3m)
NiO
Intensity (arb. units)
Q (Å^-1)
1
2
3
4
5

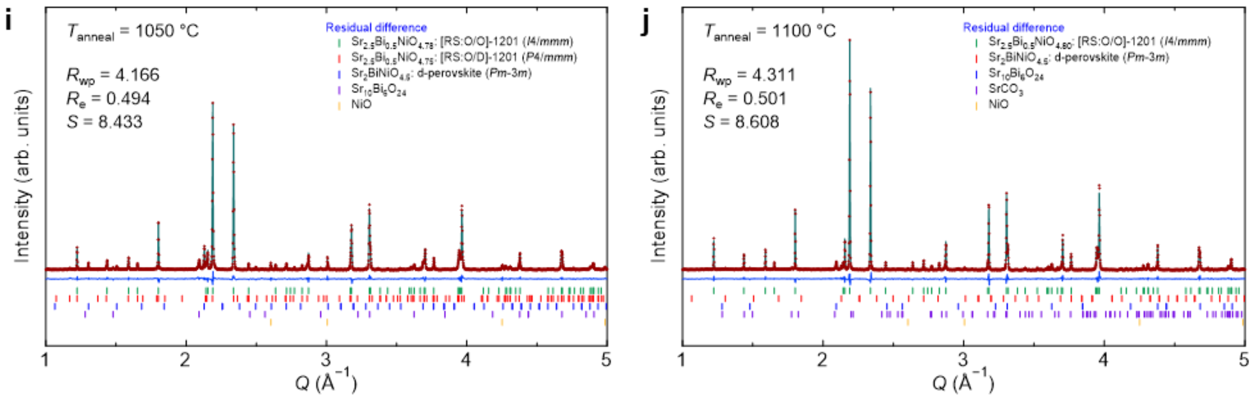

Figure S4. Synchrotron XRD patterns and the fitting results of Rietveld refinement for O-1201 with a) T_{anneal} = 100 °C, b) 200 °C, c) 300 °C, d) 400 °C, e) 500 °C, f) 600 °C, g) 950 °C, h) 1000 °C, i) 1050 °C, and j) 1100 °C, which are related to 1201-SBNO. Brown and gray curves denote the measurement data and the simulation pattern. Here, the Rietveld analysis was performed without any constraint.

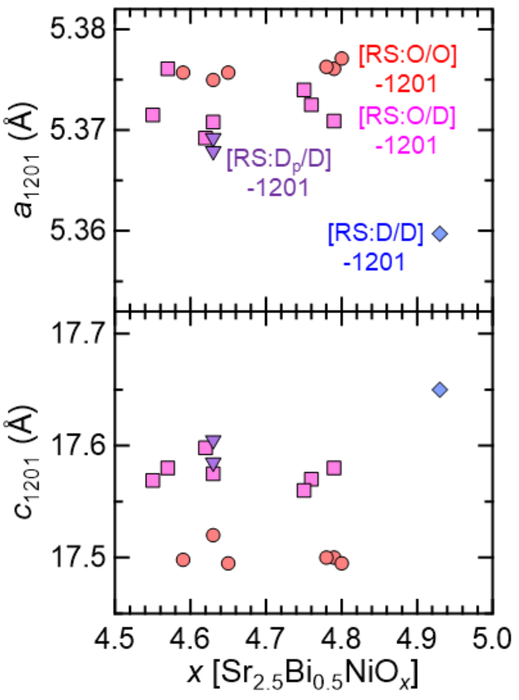

Figure S5. *a*- and *c*-axis lengths for [RS:O/O]-, [RS:O/D]-, [RS:D$_p$/D]-, and [RS:D/D]-1201 as a function of the amount of oxygen.

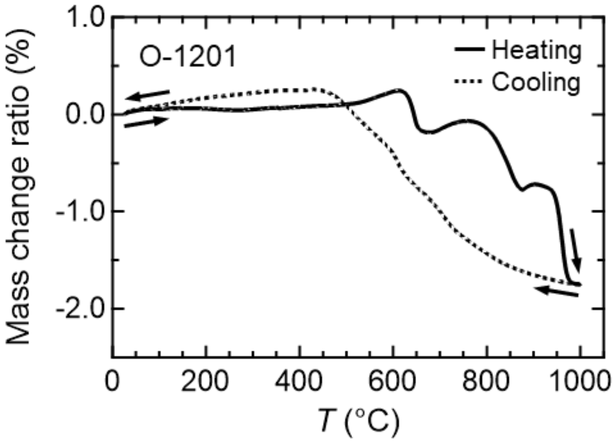

Figure S6. Temperature dependence of weight change for O-1201 during heating and cooling.

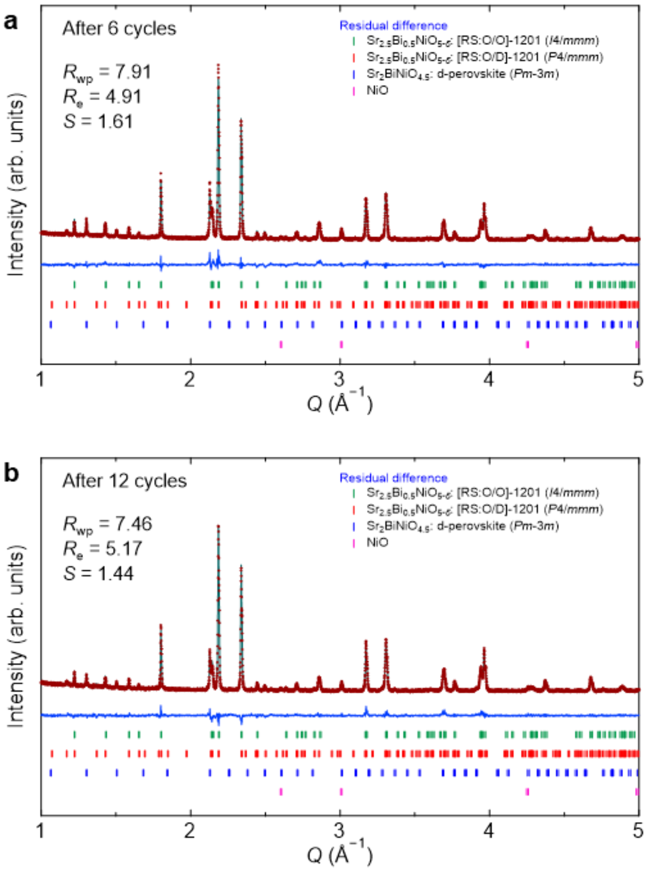

Figure S7. XRD patterns of O-1201 after a) 6 and b) 12 cycles of heating and cooling between room temperature and 1000 °C.

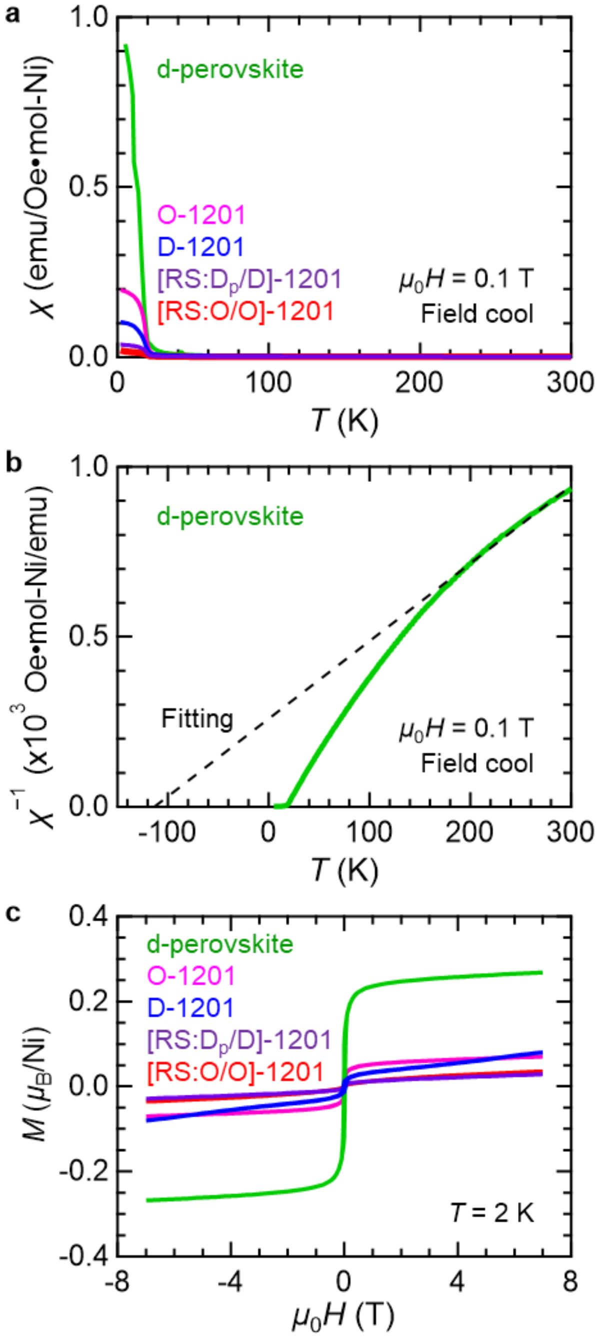

Figure S8. a) Temperature dependence of magnetic susceptibility at 0.1 T in field cooling process for for d-perovskite, [RS:O/O]-1201, O-1201, [RS:D_p/D]-1201, and D-1201. b) Temperature dependence of inverse magnetic susceptibility at 0.1 T in field cooling process for d- perovskite. c) Magnetic field dependence of magnetization at 2 K for d-perovskite, [RS:O/O]-1201, O-1201, [RS:D_p/D]-1201, and D-1201.

Figure S8a shows the temperature dependence of the magnetic susceptibility at 0.1 T in field cooling process for [RS:O/O]-1201, O-1201, [RS:D_p/D]-1201, D-1201, and d-perovskite. The d-perovskite showed a large increase in the magnetic susceptibility below 20 K (Figure S8a) with the Weiss temperature of −113 K obtained from the Curie-Weiss plot (Figure S8b), and showed a hysteretic magnetization at 2 K with the saturation magnetization of 0.24 μ_B (Figure S8c), which was significantly smaller than the Bohr magneton (2.83 μ_B) of Ni^{2+} ion (S = 1) in $Sr_2BiNiO_{4.5}$. These results suggest that the d-perovskite was a canted antiferromagnetic compound with the transition temperature of 20 K. Slight increase in magnetic susceptibility below 20 K for all the 1201-SBNO with the smaller saturation magnetization than that of d-perovskite indicates that the presence of the d-perovskite impurity phase in the 1201-SBNO, consistent with the synchrotron XRD measurement (Figure 2). No other anomaly in the magnetic susceptibility suggests that the 1201-SBNO was a paramagnetic compound, irrespective of the Sr/Bi arrangements in the rock-salt layer.

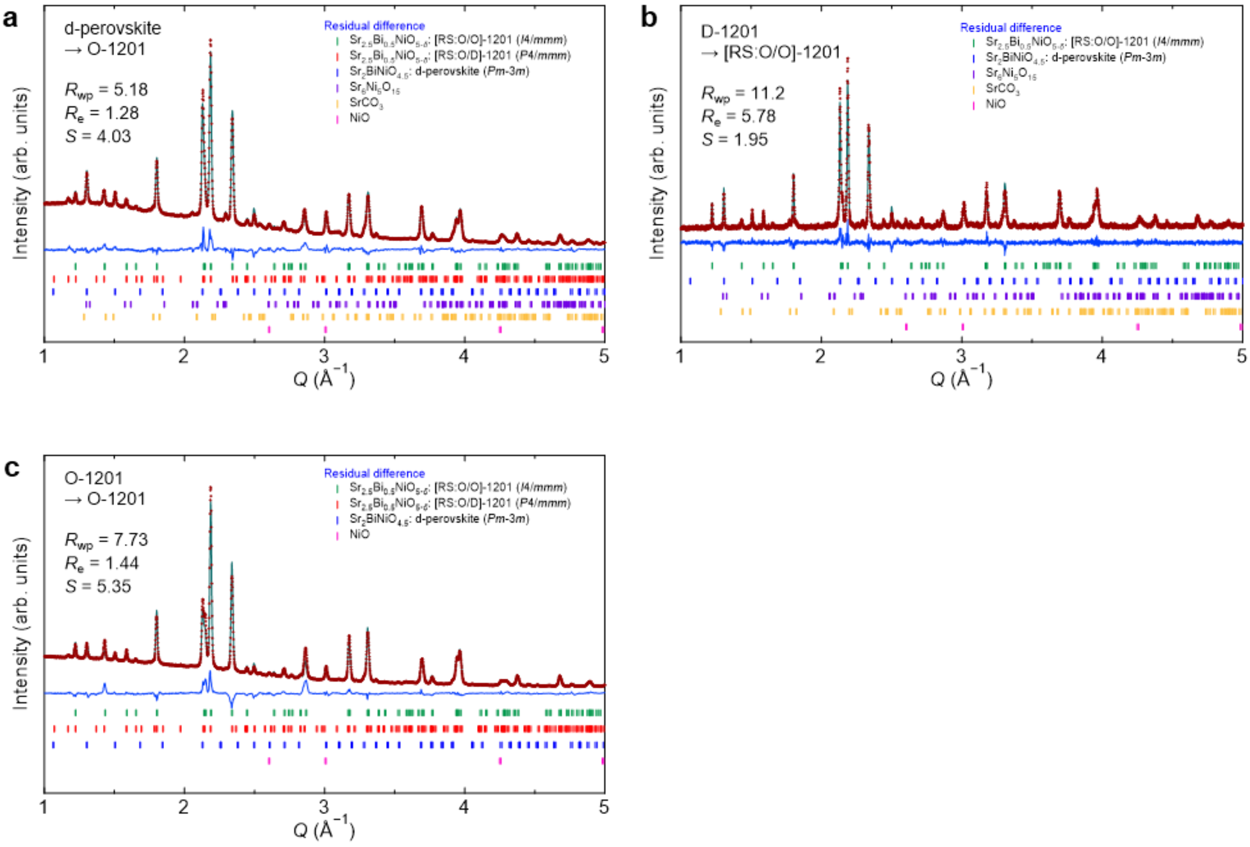

Figure S9. XRD patterns and the fitting results of Rietveld refinement for O- or [RS:O/O]-1201 after measuring the electrical resistivity above room temperature in Figure 3a for a) d-perovskite, b) D-1201, and c) O-1201. Brown and gray curves denote the measurement data and the simulation pattern. XRD patterns for a) and c) were measured by D8 Discover (Bruker AXS; Cu Kα radiation), and that for b) were measured by SmartLab (Rigaku; Cu Kα radiation), resulting in the difference for their background.

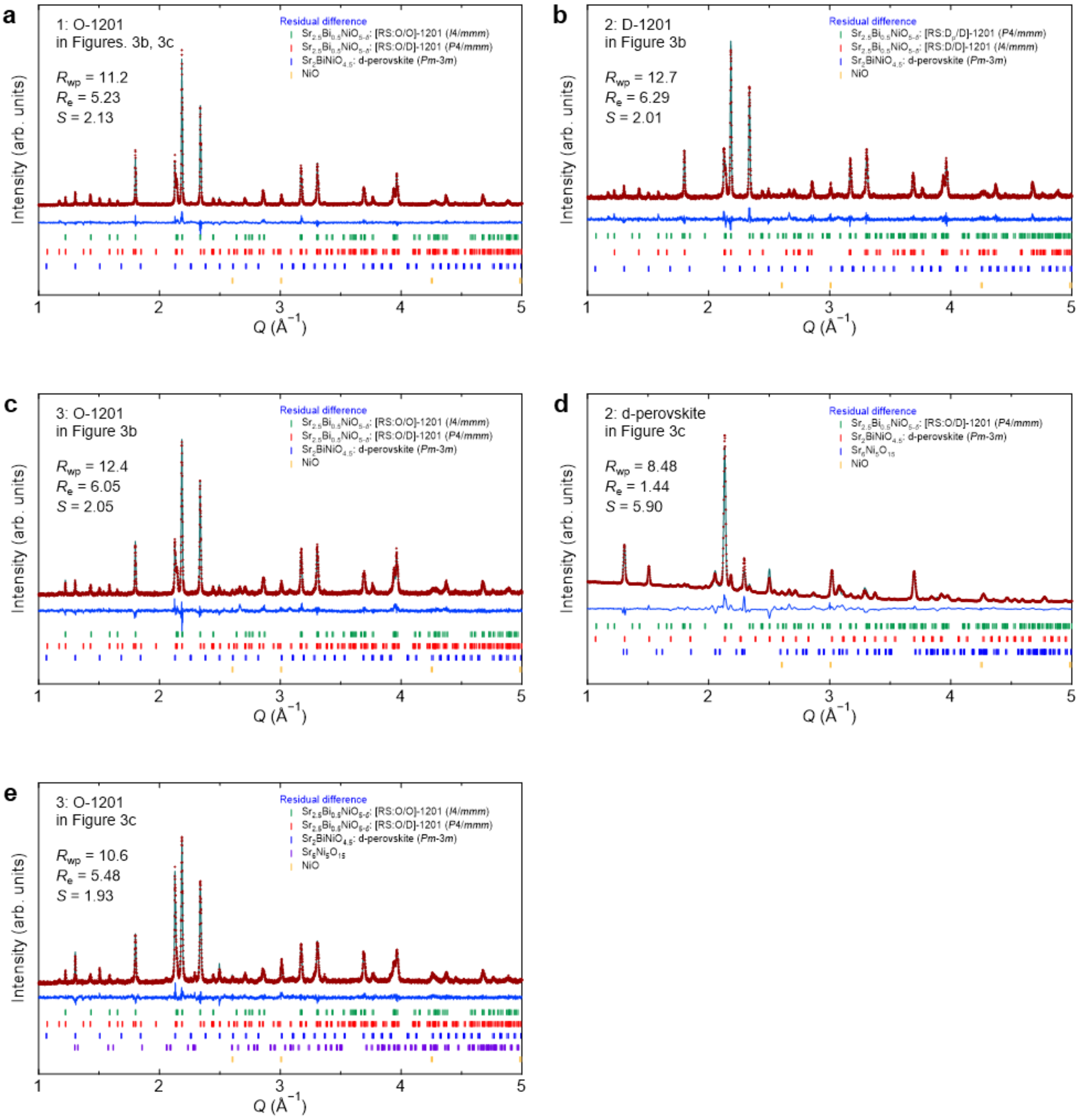

Figure S10. XRD patterns and the fitting results of Rietveld refinement for 1201-SBNO after measuring the electrical resistivity above room temperature in Figures 3b and 3c; a) 1: O-1201 in Figures 3b and 3c, b) 2: d-perovskite and c) 3: O-1201 in Figure 3b; d) 2: D-1201 and e) 3: O-1201 in Figure 3c. Brown and gray curves denote the measurement data and the simulation pattern. XRD patterns for a)–c) and e) were measured by SmartLab (Rigaku; Cu Kα radiation), and that for b) were measured by D8 Discover (Bruker AXS; Cu Kα radiation), resulting in the difference for their background.

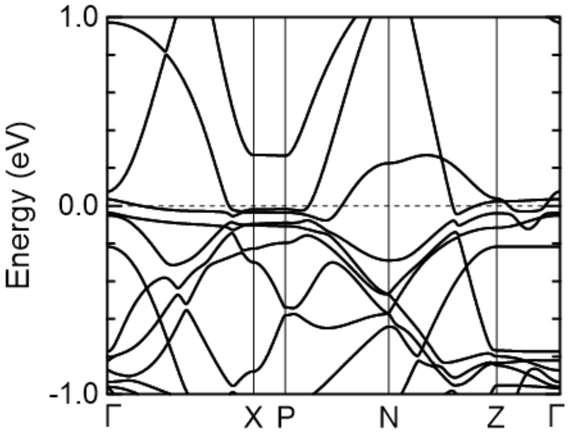

Figure S11. Band structure of [RS:O/O]-1201.

Table S1. Crystal structural parameters for O-1201 before air-annealing; a) and b) correspond to the Rietveld analysis results in Figures 1a and 1b, respectively.

a

$Sr_{2.5}Bi_{0.5}NiO_{4.95}$: [RS:O/O]-1201 (*I*4/*mmm*): *a* = 5.3768 Å, *c* = 17.500 Å

	site	*g*	*x*	*y*	*z*	*B*
Bi1	2a	1.00(0)	0	0	0	0.59(3)
Sr1	2a	0.00(0)	0	0	0	0.48(2)
Bi2	2b	0.00(0)	0	0	0.5	0.59(3)
Sr2	2b	1.00(0)	0	0	0.5	0.48(2)
Sr3	8g	1	0.5	0	0.152(0)	0.48(2)
Ni1	4e	1	0	0	0.252(0)	0.2
O1	4c	0.95(2)	0.5	0	0	1.8
O2	4e	1	0	0	0.130(1)	1.8
O3	4e	1	0	0	0.636(1)	1.8
O4	8f	1	0.25	0.25	0.25	1.8

$Sr_{2.5}Bi_{0.5}NiO_5$: [RS:O/D]-1201 (*P*4/*mmm*): *a* = 5.3713 Å, *c* = 17.566 Å

	site	*g*	*x*	*y*	*z*	*B*
Bi1	1a	0.69(2)	0	0	0	0.59(3)
Sr1	1a	0.31(2)	0	0	0	0.48(2)
Bi2	1c	0.31(2)	0.5	0.5	0	0.59(3)
Sr2	1c	0.69(2)	0.5	0.5	0	0.48(2)
Bi3	1b	0.04(2)	0	0	0.5	0.59(3)
Sr3	1b	0.96(2)	0	0	0.5	0.48(2)
Bi4	1d	0.96(2)	0.5	0.5	0.5	0.59(3)
Sr4	1d	0.04(2)	0.5	0.5	0.5	0.48(2)
Sr5	4i	1	0.5	0	0.155(1)	0.48(2)
Sr6	4i	1	0.5	0	0.351(1)	0.48(2)
Ni1	2g	1	0	0	0.249(2)	0.2
Ni2	2h	1	0.5	0.5	0.251(2)	0.2
O1	2f	1	0	0.5	0	1.8
O2	2e	1	0	0.5	0.5	1.8
O3	2g	1	0	0	0.138(6)	1.8
O4	2h	1	0.5	0.5	0.134(6)	1.8
O5	2g	1	0	0	0.376(6)	1.8
O6	2h	1	0.5	0.5	0.357(6)	1.8
O7	8r	1	0.25	0.25	0.25	1.8

b

$Sr_{2.5}Bi_{0.5}NiO_{4.95}$: [RS:O/O]-1201 (*I*4/*mmm*): *a* = 5.3768 Å, *c* = 17.500 Å

	site	*g*	*x*	*y*	*z*	*B*
Bi1	2a	1	0	0	0	0.59(3)
Sr1	2a	0	0	0	0	0.48(2)
Bi2	2b	0	0	0	0.5	0.59(3)
Sr2	2b	1	0	0	0.5	0.48(2)
Sr3	8g	1	0.5	0	0.152(0)	0.48(2)
Ni1	4e	1	0	0	0.252(0)	0.2
O1	4c	0.95(2)	0.5	0	0	1.8
O2	4e	1	0	0	0.130(1)	1.8
O3	4e	1	0	0	0.636(1)	1.8
O4	8f	1	0.25	0.25	0.25	1.8

$Sr_{2.5}Bi_{0.5}NiO_5$: [RS:O/D]-1201 (*P*4/*mmm*): *a* = 5.3713 Å, *c* = 17.566 Å

	site	*g*	*x*	*y*	*z*	*B*
Bi1	1a	0.5	0	0	0	0.59(3)
Sr1	1a	0.5	0	0	0	0.48(2)
Bi2	1c	0.5	0.5	0.5	0	0.59(3)
Sr2	1c	0.5	0.5	0.5	0	0.48(2)
Bi3	1b	0	0	0	0.5	0.59(3)
Sr3	1b	1	0	0	0.5	0.48(2)
Bi4	1d	1	0.5	0.5	0.5	0.59(3)
Sr4	1d	0	0.5	0.5	0.5	0.48(2)
Sr5	4i	1	0.5	0	0.155(1)	0.48(2)
Sr6	4i	1	0.5	0	0.351(1)	0.48(2)
Ni1	2g	1	0	0	0.249(2)	0.2
Ni2	2h	1	0.5	0.5	0.251(2)	0.2
O1	2f	1	0	0.5	0	1.8
O2	2e	1	0	0.5	0.5	1.8
O3	2g	1	0	0	0.138(6)	1.8
O4	2h	1	0.5	0.5	0.134(6)	1.8
O5	2g	1	0	0	0.376(6)	1.8
O6	2h	1	0.5	0.5	0.357(6)	1.8
O7	8r	1	0.25	0.25	0.25	1.8

Table S2. Crystal structural parameters for O-1201 with a) T_{anneal} = 100 ℃, b) 200 ℃, c) 300 ℃, d) 400 ℃, e) 500 ℃, f) 600 ℃, g) 700 ℃, h) 800 ℃, i) 900 ℃, j) 950 ℃, k) 1000 ℃, l) 1050 ℃, and m) 1100 ℃, corresponding to the Rietveld analysis results in Figures S3a–S3m, respectively. It is noted that these results are adopted in the main text.

a T_{anneal} = 100 ℃

$Sr_{2.5}Bi_{0.5}NiO_{4.79}$: [RS:O/O]-1201 (*I*4/*mmm*): *a* = 5.3761 Å, *c* = 17.500 Å

	Site	*g*	*x*	*y*	*z*	*B*
Bi1	2a	1	0	0	0	1.15(5)
Sr1	2a	0	0	0	0	0.2
Bi2	2b	0	0	0	0.5	1.15(5)
Sr2	2b	1	0	0	0.5	0.2
Sr3	8g	1	0.5	0	0.151(0)	0.2
Ni1	4e	1	0	0	0.252(0)	0.2
O1	4c	0.79(2)	0.5	0	0	1.8
O2	4e	1	0	0	0.137(2)	1.8
O3	4e	1	0	0	0.626(2)	1.8
O4	8f	1	0.25	0.25	0.25	1.8

$Sr_{2.5}Bi_{0.5}NiO_{4.57}$: [RS:O/D]-1201 (*P*4/*mmm*): *a* = 5.3761 Å, *c* = 17.580 Å

	Site	*g*	*x*	*y*	*z*	*B*
Bi1	1a	0.5	0	0	0	1.15(5)
Sr1	1a	0.5	0	0	0	0.2
Bi2	1c	0.5	0.5	0.5	0	1.15(5)
Sr2	1c	0.5	0.5	0.5	0	0.2
Bi3	1b	0	0	0	0.5	1.15(5)
Sr3	1b	1	0	0	0.5	0.2
Bi4	1d	1	0.5	0.5	0.5	1.15(5)
Sr4	1d	0	0.5	0.5	0.5	0.2
Sr5	4i	1	0.5	0	0.152(0)	0.2
Sr6	4i	1	0.5	0	0.348(0)	0.2
Ni1	2g	1	0	0	0.252(2)	0.2
Ni2	2h	1	0.5	0.5	0.249(2)	0.2
O1	2f	1	0	0.5	0	1.8
O2	2e	1	0	0.5	0.5	1.8
O3	2g	0.48(8)	0	0	0.139(7)	1.8
O4	2h	1	0.5	0.5	0.130(4)	1.8
O5	2g	1	0	0	0.366(4)	1.8
O6	2h	1	0.5	0.5	0.366(3)	1.8
O7	8r	0.92(3)	0.25	0.25	0.25	1.8

b T_{anneal} = 200 °C

$Sr_{2.5}Bi_{0.5}NiO_{4.59}$: [RS:O/O]-1201 (*I*4/*mmm*): *a* = 5.3757 Å, *c* = 17.498 Å

	site	*g*	*x*	*y*	*z*	*B*
Bi1	2a	1	0	0	0	0.80(5)
Sr1	2a	0	0	0	0	0.2
Bi2	2b	0	0	0	0.5	0.80(5)
Sr2	2b	1	0	0	0.5	0.2
Sr3	8g	1	0.5	0	0.151(0)	0.2
Ni1	4e	1	0	0	0.249(0)	0.2
O1	4c	0.59(3)	0.5	0	0	1.8
O2	4e	1	0	0	0.136(1)	1.8
O3	4e	1	0	0	0.621(1)	1.8
O4	8f	1	0.25	0.25	0.25	1.8

$Sr_{2.5}Bi_{0.5}NiO_{4.55}$: [RS:O/D]-1201 (*P*4/*mmm*): *a* = 5.3715 Å, *c* = 17.569 Å

	site	*g*	*x*	*y*	*z*	*B*
Bi1	1a	0.5	0	0	0	0.80(5)
Sr1	1a	0.5	0	0	0	0.2
Bi2	1c	0.5	0.5	0.5	0	0.80(5)
Sr2	1c	0.5	0.5	0.5	0	0.2
Bi3	1b	0	0	0	0.5	0.80(5)
Sr3	1b	1	0	0	0.5	0.2
Bi4	1d	1	0.5	0.5	0.5	0.80(5)
Sr4	1d	0	0.5	0.5	0.5	0.2
Sr5	4i	1	0.5	0	0.153(0)	0.2
Sr6	4i	1	0.5	0	0.348(0)	0.2
Ni1	2g	1	0	0	0.254(2)	0.2
Ni2	2h	1	0.5	0.5	0.247(2)	0.2
O1	2f	1	0	0.5	0	1.8
O2	2e	1	0	0.5	0.5	1.8
O3	2g	1	0	0	0.142*	1.8
O4	2h	1	0.5	0.5	0.136*	1.8
O5	2g	1	0	0	0.364*	1.8
O6	2h	1	0.5	0.5	0.363*	1.8
O7	8r	0.78(4)	0.25	0.25	0.25	1.8

* O3-, O4-, O5- , and O6- *z* were fixed with the average value for T_{anneal} = 100 °C and 300 °C, because the strong stacking fault effect was present in the XRD pattern.

c T_{anneal} = 300 °C

$Sr_{2.5}Bi_{0.5}NiO_{4.65}$: [RS:O/O]-1201 (*I*4/*mmm*): *a* = 5.3757 Å, *c* = 17.495 Å

	site	*g*	*x*	*y*	*z*	*B*
Bi1	2a	1	0	0	0	0.95(5)
Sr1	2a	0	0	0	0	0.2
Bi2	2b	0	0	0	0.5	0.95(5)
Sr2	2b	1	0	0	0.5	0.2
Sr3	8g	1	0.5	0	0.151(0)	0.2
Ni1	4e	1	0	0	0.250(0)	0.2
O1	4c	0.65(2)	0.5	0	0	1.8
O2	4e	1	0	0	0.132(1)	1.8
O3	4e	1	0	0	0.622(1)	1.8
O4	8f	1	0.25	0.25	0.25	1.8

$Sr_{2.5}Bi_{0.5}NiO_{4.76}$: [RS:O/D]-1201 (*P*4/*mmm*): *a* = 5.3725 Å, *c* = 17.570 Å

	site	*g*	*X*	*y*	*z*	*B*
Bi1	1a	0.5	0	0	0	0.95(5)
Sr1	1a	0.5	0	0	0	0.2
Bi2	1c	0.5	0.5	0.5	0	0.95(5)
Sr2	1c	0.5	0.5	0.5	0	0.2
Bi3	1b	0	0	0	0.5	0.95(5)
Sr3	1b	1	0	0	0.5	0.2
Bi4	1d	1	0.5	0.5	0.5	0.95(5)
Sr4	1d	0	0.5	0.5	0.5	0.2
Sr5	4i	1	0.5	0	0.153(0)	0.2
Sr6	4i	1	0.5	0	0.3470)	0.2
Ni1	2g	1	0	0	0.251(2)	0.2
Ni2	2h	1	0.5	0.5	0.249(2)	0.2
O1	2f	1	0	0.5	0	1.8
O2	2e	1	0	0.5	0.5	1.8
O3	2g	0.87(9)	0	0	0.147(4)	1.8
O4	2h	1	0.5	0.5	0.139(4)	1.8
O5	2g	1	0	0	0.363(4)	1.8
O6	2h	1	0.5	0.5	0.357(4)	1.8
O7	8r	0.91(3)	0.25	0.25	0.25	1.8

d T_{anneal} = 400 °C

$Sr_{2.5}Bi_{0.5}NiO_{4.63}$: [RS:O/D]-1201 (*P*4/*mmm*): *a* = 5.3708 Å, *c* = 17.575 Å

	site	*g*	*x*	*y*	*z*	*B*
Bi1	1a	0.5	0	0	0	0.95(3)
Sr1	1a	0.5	0	0	0	0.2
Bi2	1c	0.5	0.5	0.5	0	0.95(3)
Sr2	1c	0.5	0.5	0.5	0	0.2
Bi3	1b	0	0	0	0.5	0.95(3)
Sr3	1b	1	0	0	0.5	0.2
Bi4	1d	1	0.5	0.5	0.5	0.95(3)
Sr4	1d	0	0.5	0.5	0.5	0.2
Sr5	4i	1	0.5	0	0.152(0)	0.2
Sr6	4i	1	0.5	0	0.348(0)	0.2
Ni1	2g	1	0	0	0.249(1)	0.2
Ni2	2h	1	0.5	0.5	0.251(1)	0.2
O1	2f	0.66(3)	0	0.5	0	1.8
O2	2e	1	0	0.5	0.5	1.8
O3	2g	0.60(3)	0	0	0.133(2)	1.8
O4	2h	1	0.5	0.5	0.133(1)	1.8
O5	2g	1	0	0	0.378(1)	1.8
O6	2h	1	0.5	0.5	0.359(1)	1.8
O7	8r	1	0.25	0.25	0.25	1.8

e T_{anneal} = 500 °C

$Sr_{2.5}Bi_{0.5}NiO_{4.63}$: [RS:$D_p$/D]-1201 (*P*4/*mmm*): *a* = 5.3692 Å, *c* = 17.585 Å

	site	*g*	*x*	*y*	*z*	*B*
Bi1	1a	0.5	0	0	0	1.16(4)
Sr1	1a	0.5	0	0	0	0.2
Bi2	1c	0.5	0.5	0.5	0	1.16(4)
Sr2	1c	0.5	0.5	0.5	0	0.2
Bi3	1b	0.25	0	0	0.5	1.16(4)
Sr3	1b	0.75	0	0	0.5	0.2
Bi4	1d	0.75	0.5	0.5	0.5	1.16(4)
Sr4	1d	0.25	0.5	0.5	0.5	0.2
Sr5	4i	1	0.5	0	0.152(1)	0.2
Sr6	4i	1	0.5	0	0.349(1)	0.2
Ni1	2g	1	0	0	0.250(3)	0.2
Ni2	2h	1	0.5	0.5	0.253(2)	0.2
O1	2f	0.90(4)	0	0.5	0	1.8
O2	2e	1	0	0.5	0.5	1.8
O3	2g	0.57(7)	0	0	0.135(3)	1.8
O4	2h	1	0.5	0.5	0.130(2)	1.8
O5	2g	0.80(7)	0	0	0.367(2)	1.8
O6	2h	1	0.5	0.5	0.369(2)	1.8
O7	8r	1	0.25	0.25	0.25	1.8

f T_{anneal} = 600 °C

$Sr_{2.5}Bi_{0.5}NiO_{4.63}$: [RS:$D_p$/D]-1201 (*P*4/*mmm*): *a* = 5.3679 Å, *c* = 17.605 Å

	site	*g*	*x*	*y*	*z*	*B*
Bi1	1a	0.5	0	0	0	1.16
Sr1	1a	0.5	0	0	0	0.2
Bi2	1c	0.5	0.5	0.5	0	1.16
Sr2	1c	0.5	0.5	0.5	0	0.2
Bi3	1b	0.25	0	0	0.5	1.16
Sr3	1b	0.75	0	0	0.5	0.2
Bi4	1d	0.75	0.5	0.5	0.5	1.16
Sr4	1d	0.25	0.5	0.5	0.5	0.2
Sr5	4i	1	0.5	0	0.152	0.2
Sr6	4i	1	0.5	0	0.349	0.2
Ni1	2g	1	0	0	0.250	0.2
Ni2	2h	1	0.5	0.5	0.253	0.2
O1	2f	0.90	0	0.5	0	1.8
O2	2e	1	0	0.5	0.5	1.8
O3	2g	0.57	0	0	0.135	1.8
O4	2h	1	0.5	0.5	0.130	1.8
O5	2g	0.80	0	0	0.367	1.8
O6	2h	1	0.5	0.5	0.369	1.8
O7	8r	1	0.25	0.25	0.25	1.8

$Sr_{2.5}Bi_{0.5}NiO_{4.93}$: [RS:D/D]-1201 (*I*4/*mmm*): *a* = 5.3597 Å, *c* = 17.650 Å

	site	*g*	*x*	*y*	*z*	*B*
Bi1	2a	0.5	0	0	0	1.16
Sr1	2a	0.5	0	0	0	0.2
Bi2	2b	0.5	0	0	0.5	1.16
Sr2	2b	0.5	0	0	0.5	0.2
Sr3	8g	1	0.5	0	0.151(0)	0.2
Ni1	4e	1	0	0	0.252(2)	0.2
O1	4c	0.76(5)	0.5	0	0	1.8
O2	4e	1	0	0	0.134(9)	1.8
O3	4e	1	0	0	0.642(8)	1.8
O4	8f	0.76(4)	0.25	0.25	0.25	1.8
O5	16m	0.17(3)	0.25	0.25	0.054(4)	1.8

* The parameters expect for lattice constants for [RS:D_p/D]-1201 were fixed by those with T_{anneal} = 500 °C.

g T_{anneal} = 700 ℃

$Sr_2BiNiO_{4.01}$: d-perovskite (*Pm*-3*m*): *a* = 8.3241 Å

	site	*g*	*x*	*y*	*z*	*B*
Sr1	8g	1	0.230(0)	0.230(0)	0.230(0)	3.836
Ni1	1a	1	0	0	0	2.803
Ni2	3c	1	0	0.5	0.5	2.803
Bi1	3d	1	0.5	0	0	1.685
Bi2	1b	1	0.5	0.5	0.5	1.685
O1	6e	0.68(5)	0.252(8)	0	0	8.912
O2	12h	1	0.282(3)	0.5	0	8.912

h T_{anneal} = 800 ℃

$Sr_2BiNiO_{4.25}$: d-perovskite (*Pm*-3*m*): *a* = 8.3233 Å

	site	*g*	*x*	*y*	*z*	*B*
Sr1	8g	1	0.230(0)	0.230(0)	0.230(0)	3.626
Ni1	1a	1	0	0	0	2.808
Ni2	3c	1	0	0.5	0.5	2.808
Bi1	3d	1	0.5	0	0	1.720
Bi2	1b	1	0.5	0.5	0.5	1.720
O1	6e	0.84(3)	0.253(5)	0	0	9.075
O2	12h	1	0.276(2)	0.5	0	9.075

i T_{anneal} = 900 ℃

$Sr_2BiNiO_{4.50}$: d-perovskite (*Pm*-3*m*): *a* = 8.3303 Å

	site	*g*	*x*	*y*	*z*	*B*
Sr1	8g	1	0.231(0)	0.231(0)	0.231(0)	3.942
Ni1	1a	1	0	0	0	3.106
Ni2	3c	1	0	0.5	0.5	3.106
Bi1	3d	1	0.5	0	0	1.533
Bi2	1b	1	0.5	0.5	0.5	1.533
O1	6e	1	0.271(4)	0	0	6.424
O2	12h	1	0.284(2)	0.5	0	6.424

j T_{anneal} = 950 ℃

$Sr_{2.5}Bi_{0.5}NiO_{4.62}$: [RS:O/D]-1201 (*P*4/*mmm*): *a* = 5.3692 Å, *c* = 17.598 Å

	site	*g*	*x*	*y*	*z*	*B*
Bi1	1a	0.5	0	0	0	1.01(4)
Sr1	1a	0.5	0	0	0	0.2
Bi2	1c	0.5	0.5	0.5	0	1.01(4)
Sr2	1c	0.5	0.5	0.5	0	0.2
Bi3	1b	0	0	0	0.5	1.01(4)
Sr3	1b	1	0	0	0.5	0.2
Bi4	1d	1	0.5	0.5	0.5	1.01(4)
Sr4	1d	0	0.5	0.5	0.5	0.2
Sr5	4i	1	0.5	0	0.152(3)	0.2
Sr6	4i	1	0.5	0	0.349(3)	0.2
Ni1	2g	1	0	0	0.250(3)	0.2
Ni2	2h	1	0.5	0.5	0.251(4)	0.2
O1	2f	0.74(3)	0	0.5	0	1.8
O2	2e	1	0	0.5	0.5	1.8
O3	2g	0.64(5)	0	0	0.128(3)	1.8
O4	2h	1	0.5	0.5	0.134(2)	1.8
O5	2g	0.85(5)	0	0	0.376(2)	1.8
O6	2h	1	0.5	0.5	0.362(2)	1.8
O7	8r	1	0.25	0.25	0.25	1.8

k T_{anneal} = 1000 °C

$Sr_{2.5}Bi_{0.5}NiO_{4.63}$: [RS:O/O]-1201 (*I*4/*mmm*): *a* = 5.3750 Å, *c* = 17.520 Å

	site	*g*	*x*	*y*	*z*	*B*
Bi1	2a	1	0	0	0	0.98(5)
Sr1	2a	0	0	0	0	0.2
Bi2	2b	0	0	0	0.5	0.98(5)
Sr2	2b	1	0	0	0.5	0.2
Sr3	8g	1	0.5	0	0.151(0)	0.2
Ni1	4e	1	0	0	0.251(0)	0.2
O1	4c	0.69(2)	0.5	0	0	1.8
O2	4e	0.94(3)	0	0	0.134(2)	1.8
O3	4e	1	0	0	0.628(2)	1.8
O4	8f	1	0.25	0.25	0.25	1.8

$Sr_{2.5}Bi_{0.5}NiO_{4.79}$: [RS:O/D]-1201 (*P*4/*mmm*): *a* = 5.3709 Å, *c* = 17.580 Å

	site	*g*	*x*	*y*	*z*	*B*
Bi1	1a	0.5	0	0	0	0.98(5)
Sr1	1a	0.5	0	0	0	0.2
Bi2	1c	0.5	0.5	0.5	0	0.98(5)
Sr2	1c	0.5	0.5	0.5	0	0.2
Bi3	1b	0	0	0	0.5	0.98(5)
Sr3	1b	1	0	0	0.5	0.2
Bi4	1d	1	0.5	0.5	0.5	0.98(5)
Sr4	1d	0	0.5	0.5	0.5	0.2
Sr5	4i	1	0.5	0	0.152(0)	0.2
Sr6	4i	1	0.5	0	0.348(0)	0.2
Ni1	2g	1	0	0	0.250(1)	0.2
Ni2	2h	1	0.5	0.5	0.250(1)	0.2
O1	2f	1	0	0.5	0	1.8
O2	2e	1	0	0.5	0.5	1.8
O3	2g	0.58(5)	0	0	0.135(4)	1.8
O4	2h	1	0.5	0.5	0.133(3)	1.8
O5	2g	1	0	0	0.371(3)	1.8
O6	2h	1	0.5	0.5	0.362(2)	1.8
O7	8r	1	0.25	0.25	0.25	1.8

I T_{anneal} = 1050 ℃

$Sr_{2.5}Bi_{0.5}NiO_{4.78}$: [RS:O/O]-1201 (*I*4/*mmm*): *a* = 5.3763 Å, *c* = 17.500 Å

	site	*g*	*x*	*y*	*z*	*B*
Bi1	2a	1	0	0	0	0.97(4)
Sr1	2a	0	0	0	0	0.2
Bi2	2b	0	0	0	0.5	0.97(4)
Sr2	2b	1	0	0	0.5	0.2
Sr3	8g	1	0.5	0	0.152(0)	0.2
Ni1	4e	1	0	0	0.251(0)	0.2
O1	4c	0.78(2)	0.5	0	0	1.8
O2	4e	1	0	0	0.129(1)	1.8
O3	4e	1	0	0	0.629(1)	1.8
O4	8f	1	0.25	0.25	0.25	1.8

$Sr_{2.5}Bi_{0.5}NiO_{4.75}$: [RS:O/D]-1201 (*P*4/*mmm*): *a* = 5.3740 Å, *c* = 17.560 Å

	site	*g*	*x*	*Y*	*z*	*B*
Bi1	1a	0.5	0	0	0	0.97(4)
Sr1	1a	0.5	0	0	0	0.2
Bi2	1c	0.5	0.5	0.5	0	0.97(4)
Sr2	1c	0.5	0.5	0.5	0	0.2
Bi3	1b	0	0	0	0.5	0.97(4)
Sr3	1b	1	0	0	0.5	0.2
Bi4	1d	1	0.5	0.5	0.5	0.97(4)
Sr4	1d	0	0.5	0.5	0.5	0.2
Sr5	4i	1	0.5	0	0.154(2)	0.2
Sr6	4i	1	0.5	0	0.348(2)	0.2
Ni1	2g	1	0	0	0.250(2)	0.2
Ni2	2h	1	0.5	0.5	0.250(2)	0.2
O1	2f	1	0	0.5	0	1.8
O2	2e	1	0	0.5	0.5	1.8
O3	2g	1	0	0	0.139(4)	1.8
O4	2h	1	0.5	0.5	0.137(4)	1.8
O5	2g	1	0	0	0.368(4)	1.8
O6	2h	1	0.5	0.5	0.359(3)	1.8
O7	8r	0.88(3)	0.25	0.25	0.25	1.8

m T_{anneal} = 1100 ℃

$Sr_{2.5}Bi_{0.5}NiO_{4.80}$: [RS:O/O]-1201 (*I*4/*mmm*): *a* = 5.3771 Å, *c* = 17.495 Å

	site	*g*	*x*	*y*	*z*	*B*
Bi1	2a	1	0	0	0	1.00(2)
Sr1	2a	0	0	0	0	0.2
Bi2	2b	0	0	0	0.5	1.00(2)
Sr2	2b	1	0	0	0.5	0.2
Sr3	8g	1	0.5	0	0.152(0)	0.2
Ni1	4e	1	0	0	0.252(0)	0.2
O1	4c	0.87(1)	0.5	0	0	1.8
O2	4e	0.93(1)	0	0	0.133(1)	1.8
O3	4e	1	0	0	0.631(1)	1.8
O4	8f	1	0.25	0.25	0.25	1.8

Table S3. Crystal structural parameters for 1201-SBNO ($Sr_{2.5}Bi_{0.5}NiO_5$) with a) T_{anneal} = 100 °C, b) 200 °C, c) 300 °C, d) 400 °C, e) 500 °C, f) 600 °C, g) 950 °C, h) 1000 °C, i) 1050 °C, and j) 1100 °C, corresponding to the Rietveld analysis results in Figures S4a–S4j, respectively. Here, the Rietveld analysis was performed without any constraint.

a T_{anneal} = 100 ℃

$Sr_{2.5}Bi_{0.5}NiO_{4.79}$: [RS:O/O]-1201 (*I*4/*mmm*): *a* = 5.3761 Å, *c* = 17.500 Å

	site	*g*	*x*	*y*	*z*	*B*
Bi1	2a	0.96(1)	0	0	0	1.15(5)
Sr1	2a	0.04(1)	0	0	0	0.2
Bi2	2b	0.04(1)	0	0	0.5	1.15(5)
Sr2	2b	0.96(1)	0	0	0.5	0.2
Sr3	8g	1	0.5	0	0.151(0)	0.2
Ni1	4e	1	0	0	0.252(0)	0.2
O1	4c	0.79(2)	0.5	0	0	1.8
O2	4e	1	0	0	0.137(2)	1.8
O3	4e	1	0	0	0.626(2)	1.8
O4	8f	1	0.25	0.25	0.25	1.8

$Sr_{2.5}Bi_{0.5}NiO_{4.57}$: [RS:O/D]-1201 (*P*4/*mmm*): *a* = 5.3761 Å, *c* = 17.580 Å

	site	*g*	*X*	*y*	*z*	*B*
Bi1	1a	0.56(2)	0	0	0	1.15(5)
Sr1	1a	0.44(2)	0	0	0	0.2
Bi2	1c	0.44(2)	0.5	0.5	0	1.15(5)
Sr2	1c	0.56(2)	0.5	0.5	0	0.2
Bi3	1b	0.06(2)	0	0	0.5	1.15(5)
Sr3	1b	0.94(2)	0	0	0.5	0.2
Bi4	1d	0.94(2)	0.5	0.5	0.5	1.15(5)
Sr4	1d	0.06(2)	0.5	0.5	0.5	0.2
Sr5	4i	1	0.5	0	0.152(0)	0.2
Sr6	4i	1	0.5	0	0.348(0)	0.2
Ni1	2g	1	0	0	0.252(2)	0.2
Ni2	2h	1	0.5	0.5	0.249(2)	0.2
O1	2f	1	0	0.5	0	1.8
O2	2e	1	0	0.5	0.5	1.8
O3	2g	0.48(8)	0	0	0.139(7)	1.8
O4	2h	1	0.5	0.5	0.130(4)	1.8
O5	2g	1	0	0	0.366(4)	1.8
O6	2h	1	0.5	0.5	0.366(3)	1.8
O7	8r	0.92(3)	0.25	0.25	0.25	1.8

b T_{anneal} = 200 °C

$Sr_{2.5}Bi_{0.5}NiO_{4.59}$: [RS:O/O]-1201 (*I*4/*mmm*): *a* = 5.3757 Å, *c* = 17.498 Å

	site	*g*	*x*	*y*	*z*	*B*
Bi1	2a	0.97(1)	0	0	0	0.80(5)
Sr1	2a	0.03(1)	0	0	0	0.2
Bi2	2b	0.03(1)	0	0	0.5	0.80(5)
Sr2	2b	0.97(1)	0	0	0.5	0.2
Sr3	8g	1	0.5	0	0.151(0)	0.2
Ni1	4e	1	0	0	0.249(0)	0.2
O1	4c	0.59(3)	0.5	0	0	1.8
O2	4e	1	0	0	0.136(1)	1.8
O3	4e	1	0	0	0.621(1)	1.8
O4	8f	1	0.25	0.25	0.25	1.8

$Sr_{2.5}Bi_{0.5}NiO_{4.55}$: [RS:O/D]-1201 (*P*4/*mmm*): *a* = 5.3715 Å, *c* = 17.569 Å

	site	*G*	*x*	*y*	*z*	*B*
Bi1	1a	0.65(2)	0	0	0	0.80(5)
Sr1	1a	0.35(2)	0	0	0	0.2
Bi2	1c	0.35(2)	0.5	0.5	0	0.80(5)
Sr2	1c	0.65(2)	0.5	0.5	0	0.2
Bi3	1b	−0.10(2)	0	0	0.5	0.80(5)
Sr3	1b	1.10(2)	0	0	0.5	0.2
Bi4	1d	1.10(2)	0.5	0.5	0.5	0.80(5)
Sr4	1d	−0.10(2)	0.5	0.5	0.5	0.2
Sr5	4i	1	0.5	0	0.153(0)	0.2
Sr6	4i	1	0.5	0	0.348(0)	0.2
Ni1	2g	1	0	0	0.254(2)	0.2
Ni2	2h	1	0.5	0.5	0.247(2)	0.2
O1	2f	1	0	0.5	0	1.8
O2	2e	1	0	0.5	0.5	1.8
O3	2g	1	0	0	0.142*	1.8
O4	2h	1	0.5	0.5	0.136*	1.8
O5	2g	1	0	0	0.364*	1.8
O6	2h	1	0.5	0.5	0.363*	1.8
O7	8r	0.78(4)	0.25	0.25	0.25	1.8

* O3-, O4-, O5- , and O6- *z* were fixed with the average value for T_{anneal} = 100 °C and 300 °C, because the strong stacking fault effect was present in the XRD pattern.

c T_{anneal} = 300 ℃

$Sr_{2.5}Bi_{0.5}NiO_{4.65}$: [RS:O/O]-1201 (*I*4/*mmm*): *a* = 5.3757 Å, *c* = 17.495 Å

	site	*g*	*x*	*y*	*z*	*B*
Bi1	2a	0.96(1)	0	0	0	0.95(5)
Sr1	2a	0.04(1)	0	0	0	0.2
Bi2	2b	0.04(1)	0	0	0.5	0.95(5)
Sr2	2b	0.96(1)	0	0	0.5	0.2
Sr3	8g	1	0.5	0	0.151(0)	0.2
Ni1	4e	1	0	0	0.250(0)	0.2
O1	4c	0.65(2)	0.5	0	0	1.8
O2	4e	1	0	0	0.132(1)	1.8
O3	4e	1	0	0	0.622(1)	1.8
O4	8f	1	0.25	0.25	0.25	1.8

$Sr_{2.5}Bi_{0.5}NiO_{4.76}$: [RS:O/D]-1201 (*P*4/*mmm*): *a* = 5.3725 Å, *c* = 17.570 Å

	site	*g*	*X*	*y*	*z*	*B*
Bi1	1a	0.71(3)	0	0	0	0.95(5)
Sr1	1a	0.29(3)	0	0	0	0.2
Bi2	1c	0.29(3)	0.5	0.5	0	0.95(5)
Sr2	1c	0.71(3)	0.5	0.5	0	0.2
Bi3	1b	0.02(3)	0	0	0.5	0.95(5)
Sr3	1b	0.98(3)	0	0	0.5	0.2
Bi4	1d	0.98(3)	0.5	0.5	0.5	0.95(5)
Sr4	1d	0.02(3)	0.5	0.5	0.5	0.2
Sr5	4i	1	0.5	0	0.153(0)	0.2
Sr6	4i	1	0.5	0	0.3470)	0.2
Ni1	2g	1	0	0	0.251(2)	0.2
Ni2	2h	1	0.5	0.5	0.249(2)	0.2
O1	2f	1	0	0.5	0	1.8
O2	2e	1	0	0.5	0.5	1.8
O3	2g	0.87(9)	0	0	0.147(4)	1.8
O4	2h	1	0.5	0.5	0.139(4)	1.8
O5	2g	1	0	0	0.363(4)	1.8
O6	2h	1	0.5	0.5	0.357(4)	1.8
O7	8r	0.91(3)	0.25	0.25	0.25	1.8

d T_{anneal} = 400 ℃

$Sr_{2.5}Bi_{0.5}NiO_{4.63}$: [RS:O/D]-1201 (*P*4/*mmm*): *a* = 5.3708 Å, *c* = 17.575 Å

	site	*g*	*x*	*y*	*z*	*B*
Bi1	1a	0.56(1)	0	0	0	0.95(3)
Sr1	1a	0.44(1)	0	0	0	0.2
Bi2	1c	0.44(1)	0.5	0.5	0	0.95(3)
Sr2	1c	0.56(1)	0.5	0.5	0	0.2
Bi3	1b	0.08(1)	0	0	0.5	0.95(3)
Sr3	1b	0.92(1)	0	0	0.5	0.2
Bi4	1d	0.92(1)	0.5	0.5	0.5	0.95(3)
Sr4	1d	0.08(1)	0.5	0.5	0.5	0.2
Sr5	4i	1	0.5	0	0.152(0)	0.2
Sr6	4i	1	0.5	0	0.348(0)	0.2
Ni1	2g	1	0	0	0.249(1)	0.2
Ni2	2h	1	0.5	0.5	0.251(1)	0.2
O1	2f	0.66(3)	0	0.5	0	1.8
O2	2e	1	0	0.5	0.5	1.8
O3	2g	0.60(3)	0	0	0.133(2)	1.8
O4	2h	1	0.5	0.5	0.133(1)	1.8
O5	2g	1	0	0	0.378(1)	1.8
O6	2h	1	0.5	0.5	0.359(1)	1.8
O7	8r	1	0.25	0.25	0.25	1.8

e T_{anneal} = 500 °C

$Sr_{2.5}Bi_{0.5}NiO_{4.63}$: [RS:$D_p$/D]-1201 (*P*4/*mmm*): *a* = 5.3692 Å, *c* = 17.585 Å

	site	*g*	*x*	*y*	*z*	*B*
Bi1	1a	0.34(2)	0	0	0	1.16(4)
Sr1	1a	0.66(2)	0	0	0	0.2
Bi2	1c	0.66(2)	0.5	0.5	0	1.16(4)
Sr2	1c	0.34(2)	0.5	0.5	0	0.2
Bi3	1b	0.26(2)	0	0	0.5	1.16(4)
Sr3	1b	0.74(2)	0	0	0.5	0.2
Bi4	1d	0.74(2)	0.5	0.5	0.5	1.16(4)
Sr4	1d	0.26(2)	0.5	0.5	0.5	0.2
Sr5	4i	1	0.5	0	0.152(1)	0.2
Sr6	4i	1	0.5	0	0.349(1)	0.2
Ni1	2g	1	0	0	0.250(3)	0.2
Ni2	2h	1	0.5	0.5	0.253(2)	0.2
O1	2f	0.90(4)	0	0.5	0	1.8
O2	2e	1	0	0.5	0.5	1.8
O3	2g	0.57(7)	0	0	0.135(3)	1.8
O4	2h	1	0.5	0.5	0.130(2)	1.8
O5	2g	0.80(7)	0	0	0.367(2)	1.8
O6	2h	1	0.5	0.5	0.369(2)	1.8
O7	8r	1	0.25	0.25	0.25	1.8

f T_{anneal} = 600 ℃

$Sr_{2.5}Bi_{0.5}NiO_{4.21}$: [RS:$D_p$/D]-1201 (*P*4/*mmm*): *a* = 5.3679 Å, *c* = 17.605 Å

	site	*g*	*x*	*Y*	*z*	*B*
Bi1	1a	0.48(3)	0	0	0	1.30(11)
Sr1	1a	0.52(3)	0	0	0	0.24(5)
Bi2	1c	0.52(3)	0.5	0.5	0	1.30(11)
Sr2	1c	0.48(3)	0.5	0.5	0	0.24(5)
Bi3	1b	0.23(3)	0	0	0.5	1.30(11)
Sr3	1b	0.77(3)	0	0	0.5	0.24(5)
Bi4	1d	0.77(3)	0.5	0.5	0.5	1.30(11)
Sr4	1d	0.23(3)	0.5	0.5	0.5	0.24(5)
Sr5	4i	1	0.5	0	0.152(2)	0.24(5)
Sr6	4i	1	0.5	0	0.348(2)	0.24(5)
Ni1	2g	1	0	0	0.249(4)	0.2
Ni2	2h	1	0.5	0.5	0.255(2)	0.2
O1	2f	0.59(6)	0	0.5	0	1.8
O2	2e	1	0	0.5	0.5	1.8
O3	2g	0.58(6)	0	0	0.135(5)	1.8
O4	2h	1	0.5	0.5	0.136(3)	1.8
O5	2g	0.65(6)	0	0	0.363(5)	1.8
O6	2h	1	0.5	0.5	0.361(3)	1.8
O7	8r	0.90(3)	0.25	0.25	0.25	1.8

$Sr_{2.5}Bi_{0.5}NiO_{5.28}$: [RS:D/D]-1201 (*I*4/*mmm*): *a* = 5.3597 Å, *c* = 17.650 Å

	site	*g*	*x*	*y*	*z*	*B*
Bi1	2a	0.45(11)	0	0	0	1.30(11)
Sr1	2a	0.55(11)	0	0	0	0.24(5)
Bi2	2b	0.55(11)	0	0	0.5	1.30(11)
Sr2	2b	0.45(11)	0	0	0.5	0.24(5)
Sr3	8g	1	0.5	0	0.151(0)	0.24(5)
Ni1	4e	1	0	0	0.253(3)	0.2
O1	4c	0.95(5)	0.5	0	0	1.8
O2	4e	1	0	0	0.128(3)	1.8
O3	4e	1	0	0	0.638(3)	1.8
O4	8f	0.85(4)	0.25	0.25	0.25	1.8
O5	16m	0.16(3)	0.25	0.25	0.060(6)	1.8

g T_{anneal} = 950 ℃

$Sr_{2.5}Bi_{0.5}NiO_{4.62}$: [RS:O/D]-1201 (*P*4/*mmm*): *a* = 5.3692 Å, *c* = 17.598 Å

	site	*g*	*x*	*y*	*z*	*B*
Bi1	1a	0.56(1)	0	0	0	1.01(4)
Sr1	1a	0.44(1)	0	0	0	0.2
Bi2	1c	0.44(1)	0.5	0.5	0	1.01(4)
Sr2	1c	0.56(1)	0.5	0.5	0	0.2
Bi3	1b	0.08(1)	0	0	0.5	1.01(4)
Sr3	1b	0.92(1)	0	0	0.5	0.2
Bi4	1d	0.92(1)	0.5	0.5	0.5	1.01(4)
Sr4	1d	0.08(1)	0.5	0.5	0.5	0.2
Sr5	4i	1	0.5	0	0.152(3)	0.2
Sr6	4i	1	0.5	0	0.349(3)	0.2
Ni1	2g	1	0	0	0.250(3)	0.2
Ni2	2h	1	0.5	0.5	0.251(4)	0.2
O1	2f	0.74(3)	0	0.5	0	1.8
O2	2e	1	0	0.5	0.5	1.8
O3	2g	0.64(5)	0	0	0.128(3)	1.8
O4	2h	1	0.5	0.5	0.134(2)	1.8
O5	2g	0.85(5)	0	0	0.376(2)	1.8
O6	2h	1	0.5	0.5	0.362(2)	1.8
O7	8r	1	0.25	0.25	0.25	1.8

h T_{anneal} = 1000 °C

$Sr_{2.5}Bi_{0.5}NiO_{4.63}$: [RS:O/O]-1201 (*I*4/*mmm*): *a* = 5.3750 Å, *c* = 17.520 Å

	site	*g*	*x*	*y*	*z*	*B*
Bi1	2a	0.98(1)	0	0	0	0.98(5)
Sr1	2a	0.02(1)	0	0	0	0.2
Bi2	2b	0.02(1)	0	0	0.5	0.98(5)
Sr2	2b	0.98(1)	0	0	0.5	0.2
Sr3	8g	1	0.5	0	0.151(0)	0.2
Ni1	4e	1	0	0	0.251(0)	0.2
O1	4c	0.69(2)	0.5	0	0	1.8
O2	4e	0.94(3)	0	0	0.134(2)	1.8
O3	4e	1	0	0	0.628(2)	1.8
O4	8f	1	0.25	0.25	0.25	1.8

$Sr_{2.5}Bi_{0.5}NiO_{4.79}$: [RS:O/D]-1201 (*P*4/*mmm*): *a* = 5.3709 Å, *c* = 17.580 Å

	site	*g*	*x*	*y*	*z*	*B*
Bi1	1a	0.54(2)	0	0	0	0.98(5)
Sr1	1a	0.46(2)	0	0	0	0.2
Bi2	1c	0.46(2)	0.5	0.5	0	0.98(5)
Sr2	1c	0.54(2)	0.5	0.5	0	0.2
Bi3	1b	0.08(2)	0	0	0.5	0.98(5)
Sr3	1b	0.92(2)	0	0	0.5	0.2
Bi4	1d	0.92(2)	0.5	0.5	0.5	0.98(5)
Sr4	1d	0.08(2)	0.5	0.5	0.5	0.2
Sr5	4i	1	0.5	0	0.152(0)	0.2
Sr6	4i	1	0.5	0	0.348(0)	0.2
Ni1	2g	1	0	0	0.250(1)	0.2
Ni2	2h	1	0.5	0.5	0.250(1)	0.2
O1	2f	1	0	0.5	0	1.8
O2	2e	1	0	0.5	0.5	1.8
O3	2g	0.58(5)	0	0	0.135(4)	1.8
O4	2h	1	0.5	0.5	0.133(3)	1.8
O5	2g	1	0	0	0.371(3)	1.8
O6	2h	1	0.5	0.5	0.362(2)	1.8
O7	8r	1	0.25	0.25	0.25	1.8

i T_{anneal} = 1050 ℃

$Sr_{2.5}Bi_{0.5}NiO_{4.78}$: [RS:O/O]-1201 (*I*4/*mmm*): *a* = 5.3763 Å, *c* = 17.500 Å

	site	*g*	*x*	*y*	*z*	*B*
Bi1	2a	1.00(0)	0	0	0	0.97(4)
Sr1	2a	0.00(0)	0	0	0	0.2
Bi2	2b	0.00(0)	0	0	0.5	0.97(4)
Sr2	2b	1.00(0)	0	0	0.5	0.2
Sr3	8g	1	0.5	0	0.152(0)	0.2
Ni1	4e	1	0	0	0.251(0)	0.2
O1	4c	0.78(2)	0.5	0	0	1.8
O2	4e	1	0	0	0.129(1)	1.8
O3	4e	1	0	0	0.629(1)	1.8
O4	8f	1	0.25	0.25	0.25	1.8

$Sr_{2.5}Bi_{0.5}NiO_{4.75}$: [RS:O/D]-1201 (*P*4/*mmm*): *a* = 5.3740 Å, *c* = 17.560 Å

	site	*g*	*x*	*Y*	*z*	*B*
Bi1	1a	0.60(2)	0	0	0	0.97(4)
Sr1	1a	0.40(2)	0	0	0	0.2
Bi2	1c	0.40(2)	0.5	0.5	0	0.97(4)
Sr2	1c	0.60(2)	0.5	0.5	0	0.2
Bi3	1b	0.10(3)	0	0	0.5	0.97(4)
Sr3	1b	0.90(3)	0	0	0.5	0.2
Bi4	1d	0.90(3)	0.5	0.5	0.5	0.97(4)
Sr4	1d	0.10(3)	0.5	0.5	0.5	0.2
Sr5	4i	1	0.5	0	0.154(2)	0.2
Sr6	4i	1	0.5	0	0.348(2)	0.2
Ni1	2g	1	0	0	0.250(2)	0.2
Ni2	2h	1	0.5	0.5	0.250(2)	0.2
O1	2f	1	0	0.5	0	1.8
O2	2e	1	0	0.5	0.5	1.8
O3	2g	1	0	0	0.139(4)	1.8
O4	2h	1	0.5	0.5	0.137(4)	1.8
O5	2g	1	0	0	0.368(4)	1.8
O6	2h	1	0.5	0.5	0.359(3)	1.8
O7	8r	0.88(3)	0.25	0.25	0.25	1.8

j T_{anneal} = 1100 ℃

$Sr_{2.5}Bi_{0.5}NiO_{4.80}$: [RS:O/O-1201] (*I*4/*mmm*): *a* = 5.3771 Å, *c* = 17.495 Å

	site	*g*	*x*	*y*	*z*	*B*
Bi1	2a	0.99(0)	0	0	0	1.00(2)
Sr1	2a	0.01(0)	0	0	0	0.2
Bi2	2b	0.01(0)	0	0	0.5	1.00(2)
Sr2	2b	0.99(0)	0	0	0.5	0.2
Sr3	8g	1	0.5	0	0.152(0)	0.2
Ni1	4e	1	0	0	0.252(0)	0.2
O1	4c	0.87(1)	0.5	0	0	1.8
O2	4e	0.93(1)	0	0	0.133(1)	1.8
O3	4e	1	0	0	0.631(1)	1.8
O4	8f	1	0.25	0.25	0.25	1.8

Table S4. Phase fractions of [RS:O/O]-1201, [RS:O/D]-1201, [RS:D_p/D]-1201, [RS:D/D]-1201, and d-perovskite without air-annealing and with T_{anneal} = 100–1100 °C., calculated by Rietveld analysis in Figures. S2 and S3 (R_{wp}: R-factor, R_e: expected R-factor, S: goodness-of-fit indicator).

T_{anneal} (°C)	Before anneal	100	200	300	400	500	600	700	800	900	950	1000	1050	1100
[RS:O/O]-1201 (mol%)	61	52	53	55	0	0	0	0	0	0	0	43	61	99
[RS:O/D]-1201 (mol%)	36	44	43	42	98	0	0	0	0	0	76	45	34	0
[RS:D_p/D]-1201 (mol%)	0	0	0	0	0	96	56	0	0	0	0	0	0	0
[RS:D_p/D]-1201 (mol%)	0	0	0	0	0	0	37	0	0	0	0	0	0	0
d-perovskite (mol%)	3	4	4	3	2	4	7	100	100	100	24	12	5	1
R_{wp}	6.48	3.95	4.59	4.05	4.31	4.86	4.65	5.31	5.95	7.16	4.34	4.10	4.18	4.32
R_e	0.917	0.503	0.480	0.470	0.497	0.503	0.491	0.490	0.503	0.597	0.491	0.500	0.494	0.501
S	7.06	7.85	9.56	8.60	8.69	9.66	9.45	10.8	11.8	14.4	8.84	8.19	8.46	8.63

Table S5. Chemical composition of O-1201 without air-annealing and with T_{anneal} = 100–1100 °C. It is noted that the nominal composition is Sr:Ni:Bi = 62.5:12.5:25.0.

T_{anneal} (°C)	Before anneal	100	200	300	400	500	600	700	800	900	950	1000	1050	1100
Sr (at%)	63	63	63	63	65	65	63	62	61	60	60	62	63	61
Bi (at%)	12	14	13	13	13	12	13	11	14	14	14	13	12	14
Ni (at%)	26	23	24	24	22	23	24	27	25	26	26	25	25	25

Table S6. Crystal structural parameters for O-1201 after a) 6 and b) 12 cycles of heating and cooling between room temperature and 1000 °C, corresponding to the Rietveld analysis results in Figures S7a and S7b, respectively.

a O-1201 after 6 cycles

$Sr_{2.5}Bi_{0.5}NiO_{4.63}$: [RS:O/O]-1201 (*I*4/*mmm*): *a* = 5.3743 Å, *c* = 17.545 Å

	Site	*g*	*x*	*y*	*z*	*B*
Bi1	2a	1	0	0	0	0.979
Sr1	2a	0	0	0	0	0.2
Bi2	2b	0	0	0	0.5	0.979
Sr2	2b	1	0	0	0.5	0.2
Sr3	8g	1	0.5	0	0.151	0.2
Ni1	4e	1	0	0	0.251	0.2
O1	4c	0.69	0.5	0	0	1.8
O2	4e	0.94	0	0	0.134	1.8
O3	4e	1	0	0	0.628	1.8
O4	8f	1	0.25	0.25	0.25	1.8

$Sr_{2.5}Bi_{0.5}NiO_{4.79}$: [RS:O/D]-1201 (*P*4/*mmm*): *a* = 5.3698 Å, *c* = 17.597 Å

	Site	*g*	*x*	*y*	*z*	*B*
Bi1	1a	0.5	0	0	0	0.979
Sr1	1a	0.5	0	0	0	0.2
Bi2	1c	0.5	0.5	0.5	0	0.979
Sr2	1c	0.5	0.5	0.5	0	0.2
Bi3	1b	0	0	0	0.5	0.979
Sr3	1b	1	0	0	0.5	0.2
Bi4	1d	1	0.5	0.5	0.5	0.979
Sr4	1d	0	0.5	0.5	0.5	0.2
Sr5	4i	1	0.5	0	0.152	0.2
Sr6	4i	1	0.5	0	0.348	0.2
Ni1	2g	1	0	0	0.250	0.2
Ni2	2h	1	0.5	0.5	0.250	0.2
O1	2f	1	0	0.5	0	1.8
O2	2e	1	0	0.5	0.5	1.8
O3	2g	0.58	0	0	0.135	1.8
O4	2h	1	0.5	0.5	0.133	1.8
O5	2g	1	0	0	0.371	1.8
O6	2h	1	0.5	0.5	0.362	1.8
O7	8r	1	0.25	0.25	0.25	1.8

b O-1201 after 12 cycles

$Sr_{2.5}Bi_{0.5}NiO_{4.63}$: [RS:O/O]-1201 (*I*4/*mmm*): *a* = 5.3731 Å, *c* = 17.533 Å

	Site	*g*	*x*	*y*	*z*	*B*
Bi1	2a	1	0	0	0	0.979
Sr1	2a	0	0	0	0	0.2
Bi2	2b	0	0	0	0.5	0.979
Sr2	2b	1	0	0	0.5	0.2
Sr3	8g	1	0.5	0	0.151	0.2
Ni1	4e	1	0	0	0.251	0.2
O1	4c	0.69	0.5	0	0	1.8
O2	4e	0.94	0	0	0.134	1.8
O3	4e	1	0	0	0.628	1.8
O4	8f	1	0.25	0.25	0.25	1.8

$Sr_{2.5}Bi_{0.5}NiO_{4.79}$: [RS:O/D]-1201 (*P*4/*mmm*): *a* = 5.3689 Å, *c* = 17.586 Å

	Site	*g*	*x*	*y*	*z*	*B*
Bi1	1a	0.5	0	0	0	0.979
Sr1	1a	0.5	0	0	0	0.2
Bi2	1c	0.5	0.5	0.5	0	0.979
Sr2	1c	0.5	0.5	0.5	0	0.2
Bi3	1b	0	0	0	0.5	0.979
Sr3	1b	1	0	0	0.5	0.2
Bi4	1d	1	0.5	0.5	0.5	0.979
Sr4	1d	0	0.5	0.5	0.5	0.2
Sr5	4i	1	0.5	0	0.152	0.2
Sr6	4i	1	0.5	0	0.348	0.2
Ni1	2g	1	0	0	0.250	0.2
Ni2	2h	1	0.5	0.5	0.250	0.2
O1	2f	1	0	0.5	0	1.8
O2	2e	1	0	0.5	0.5	1.8
O3	2g	0.58	0	0	0.135	1.8
O4	2h	1	0.5	0.5	0.133	1.8
O5	2g	1	0	0	0.371	1.8
O6	2h	1	0.5	0.5	0.362	1.8
O7	8r	1	0.25	0.25	0.25	1.8

Table S7. Phase fractions of [RS:O/O]-1201, [RS:O/D]-1201, [RS:D_p/D]-1201, [RS:D/D]-1201, and d-perovskite in O-1201 before cycling, and after 6 and 12 cycles of heating and cooling between room temperature and 1000 °C.

	before	After 6 cycles	After 12 cycles
[RS:O/O]-1201 (mol%)	43	36	31
[RS:O/D]-1201 (mol%)	45	52	60
[RS:D_p/D]-1201 (mol%)	0	0	0
[RS:D/D]-1201 (mol%)	0	0	0
d-perovskite (mol%)	12	12	9

Table S8. Crystal structural parameters for O- or [RS:O/O]-1201 after measuring the electrical resistivity above room temperature in Figure 3a for a) d-perovskite, b) D-1201, and c) O-1201, corresponding to the Rietveld analysis results in Figures S9a–S9c, respectively.

a O-1201 in Figure S9a

$Sr_{2.5}Bi_{0.5}NiO_{4.63}$: [RS:O/O]-1201 ($I4/mmm$): a = 5.3696 Å, c = 17.569 Å

	Site	*g*	*x*	*y*	*z*	*B*
Bi1	2a	1	0	0	0	0.979
Sr1	2a	0	0	0	0	0.2
Bi2	2b	0	0	0	0.5	0.979
Sr2	2b	1	0	0	0.5	0.2
Sr3	8g	1	0.5	0	0.151	0.2
Ni1	4e	1	0	0	0.251	0.2
O1	4c	0.69	0.5	0	0	1.8
O2	4e	0.94	0	0	0.134	1.8
O3	4e	1	0	0	0.628	1.8
O4	8f	1	0.25	0.25	0.25	1.8

$Sr_{2.5}Bi_{0.5}NiO_{4.79}$: [RS:O/D]-1201 ($P4/mmm$): a = 5.3612 Å, c = 17.618 Å

	Site	*g*	*x*	*y*	*z*	*B*
Bi1	1a	0.5	0	0	0	0.979
Sr1	1a	0.5	0	0	0	0.2
Bi2	1c	0.5	0.5	0.5	0	0.979
Sr2	1c	0.5	0.5	0.5	0	0.2
Bi3	1b	0	0	0	0.5	0.979
Sr3	1b	1	0	0	0.5	0.2
Bi4	1d	1	0.5	0.5	0.5	0.979
Sr4	1d	0	0.5	0.5	0.5	0.2
Sr5	4i	1	0.5	0	0.152	0.2
Sr6	4i	1	0.5	0	0.348	0.2
Ni1	2g	1	0	0	0.250	0.2
Ni2	2h	1	0.5	0.5	0.250	0.2
O1	2f	1	0	0.5	0	1.8
O2	2e	1	0	0.5	0.5	1.8
O3	2g	0.58	0	0	0.135	1.8
O4	2h	1	0.5	0.5	0.133	1.8
O5	2g	1	0	0	0.371	1.8
O6	2h	1	0.5	0.5	0.362	1.8
O7	8r	1	0.25	0.25	0.25	1.8

b [RS:O/O]-1201 in Figure S9b

$Sr_{2.5}Bi_{0.5}NiO_{4.63}$: [RS:O/O]-1201 (*I*4/*mmm*): *a* = 5.3742 Å, *c* = 17.539 Å

	Site	*g*	*x*	*y*	*z*	*B*
Bi1	2a	1	0	0	0	0.979
Sr1	2a	0	0	0	0	0.2
Bi2	2b	0	0	0	0.5	0.979
Sr2	2b	1	0	0	0.5	0.2
Sr3	8g	1	0.5	0	0.151	0.2
Ni1	4e	1	0	0	0.251	0.2
O1	4c	0.69	0.5	0	0	1.8
O2	4e	0.94	0	0	0.134	1.8
O3	4e	1	0	0	0.628	1.8
O4	8f	1	0.25	0.25	0.25	1.8

c O-1201 in Figure S9c

$Sr_{2.5}Bi_{0.5}NiO_{4.63}$: [RS:O/O]-1201 (*I*4/*mmm*): *a* = 5.3719 Å, *c* = 17.530 Å

	Site	*g*	*x*	*y*	*z*	*B*
Bi1	2a	1	0	0	0	0.979
Sr1	2a	0	0	0	0	0.2
Bi2	2b	0	0	0	0.5	0.979
Sr2	2b	1	0	0	0.5	0.2
Sr3	8g	1	0.5	0	0.151	0.2
Ni1	4e	1	0	0	0.251	0.2
O1	4c	0.69	0.5	0	0	1.8
O2	4e	0.94	0	0	0.134	1.8
O3	4e	1	0	0	0.628	1.8
O4	8f	1	0.25	0.25	0.25	1.8

$Sr_{2.5}Bi_{0.5}NiO_{4.79}$: O_p-1201 (*P*4/*mmm*): *a* = 5.3707 Å, *c* = 17.597 Å

	Site	*g*	*x*	*y*	*z*	*B*
Bi1	1a	0.5	0	0	0	0.979
Sr1	1a	0.5	0	0	0	0.2
Bi2	1c	0.5	0.5	0.5	0	0.979
Sr2	1c	0.5	0.5	0.5	0	0.2
Bi3	1b	0	0	0	0.5	0.979
Sr3	1b	1	0	0	0.5	0.2
Bi4	1d	1	0.5	0.5	0.5	0.979
Sr4	1d	0	0.5	0.5	0.5	0.2
Sr5	4i	1	0.5	0	0.152	0.2
Sr6	4i	1	0.5	0	0.348	0.2
Ni1	2g	1	0	0	0.250	0.2
Ni2	2h	1	0.5	0.5	0.250	0.2
O1	2f	1	0	0.5	0	1.8
O2	2e	1	0	0.5	0.5	1.8
O3	2g	0.58	0	0	0.135	1.8
O4	2h	1	0.5	0.5	0.133	1.8
O5	2g	1	0	0	0.371	1.8
O6	2h	1	0.5	0.5	0.362	1.8
O7	8r	1	0.25	0.25	0.25	1.8

Table S9. Crystal structural parameters for 1201-SBNO after measuring the electrical resistivity above room temperature in Figures 3b and 3c; a) 1: O-1201 in Figures 3b and 3c, b) 2: d-perovskite and c) 3: O-1201 in Figure 3b; d) 2: D-1201 and e) 3: O-1201 in Figure 3c, corresponding to the Rietveld analysis results in Figures S10a–S10e respectively.

a O-1201 in Figure S10a

$Sr_{2.5}Bi_{0.5}NiO_{4.63}$: [RS:O/O]-1201 (*I*4/*mmm*): *a* = 5.3773 Å, *c* = 17.545 Å

	Site	*g*	*x*	*y*	*z*	*B*
Bi1	2a	1	0	0	0	0.979
Sr1	2a	0	0	0	0	0.2
Bi2	2b	0	0	0	0.5	0.979
Sr2	2b	1	0	0	0.5	0.2
Sr3	8g	1	0.5	0	0.151	0.2
Ni1	4e	1	0	0	0.251	0.2
O1	4c	0.69	0.5	0	0	1.8
O2	4e	0.94	0	0	0.134	1.8
O3	4e	1	0	0	0.628	1.8
O4	8f	1	0.25	0.25	0.25	1.8

$Sr_{2.5}Bi_{0.5}NiO_{4.79}$: [RS:O/D]-1201 (*P*4/*mmm*): *a* = 5.3698 Å, *c* = 17.591 Å

	Site	*g*	*x*	*y*	*z*	*B*
Bi1	1a	0.5	0	0	0	0.979
Sr1	1a	0.5	0	0	0	0.2
Bi2	1c	0.5	0.5	0.5	0	0.979
Sr2	1c	0.5	0.5	0.5	0	0.2
Bi3	1b	0	0	0	0.5	0.979
Sr3	1b	1	0	0	0.5	0.2
Bi4	1d	1	0.5	0.5	0.5	0.979
Sr4	1d	0	0.5	0.5	0.5	0.2
Sr5	4i	1	0.5	0	0.152	0.2
Sr6	4i	1	0.5	0	0.348	0.2
Ni1	2g	1	0	0	0.250	0.2
Ni2	2h	1	0.5	0.5	0.250	0.2
O1	2f	1	0	0.5	0	1.8
O2	2e	1	0	0.5	0.5	1.8
O3	2g	0.58	0	0	0.135	1.8
O4	2h	1	0.5	0.5	0.133	1.8
O5	2g	1	0	0	0.371	1.8
O6	2h	1	0.5	0.5	0.362	1.8
O7	8r	1	0.25	0.25	0.25	1.8

b D-1201 in Figure S10b

$Sr_{2.5}Bi_{0.5}NiO_{4.18}$: [RS:$D_p$/D]-1201 (*P*4/*mmm*): *a* = 5.3750 Å, *c* = 17.605 Å

	site	*g*	*x*	*y*	*z*	*B*
Bi1	1a	0.5	0	0	0	1.293
Sr1	1a	0.5	0	0	0	0.202
Bi2	1c	0.5	0.5	0.5	0	1.293
Sr2	1c	0.5	0.5	0.5	0	0.202
Bi3	1b	0.25	0	0	0.5	1.293
Sr3	1b	0.75	0	0	0.5	0.202
Bi4	1d	0.75	0.5	0.5	0.5	1.293
Sr4	1d	0.25	0.5	0.5	0.5	0.2
Sr5	4i	1	0.5	0	0.152	0.2
Sr6	4i	1	0.5	0	0.348	0.2
Ni1	2g	1	0	0	0.250	0.2
Ni2	2h	1	0.5	0.5	0.255	0.2
O1	2f	0.58	0	0.5	0	1.8
O2	2e	1	0	0.5	0.5	1.8
O3	2g	0.60	0	0	0.135	1.8
O4	2h	1	0.5	0.5	0.136	1.8
O5	2g	0.63	0	0	0.362	1.8
O6	2h	1	0.5	0.5	0.363	1.8
O7	8r	0.89	0.25	0.25	0.25	1.8

$Sr_{2.5}Bi_{0.5}NiO_{5.29}$: [RS:D/D]-1201 (*I*4/*mmm*): *a* = 5.3700 Å, *c* = 17.650 Å

	site	*g*	*x*	*y*	*z*	*B*
Bi1	2a	0.5	0	0	0	1.298
Sr1	2a	0.5	0	0	0	0.237
Bi2	2b	0.5	0	0	0.5	1.298
Sr2	2b	0.5	0	0	0.5	0.237
Sr3	8g	1	0.5	0	0.151	0.237
Ni1	4e	1	0	0	0.253	0.2
O1	4c	0.96	0.5	0	0	1.8
O2	4e	1	0	0	0.128	1.8
O3	4e	1	0	0	0.638	1.8
O4	8f	0.85	0.25	0.25	0.25	1.8
O5	16m	0.16	0.25	0.25	0.060	1.8

c O-1201 in Figure S10c

$Sr_{2.5}Bi_{0.5}NiO_{4.63}$: [RS:O/O]-1201 (*I*4/*mmm*): *a* = 5.3764 Å, *c* = 17.522 Å

	Site	*g*	*x*	*y*	*z*	*B*
Bi1	2a	1	0	0	0	0.979
Sr1	2a	0	0	0	0	0.2
Bi2	2b	0	0	0	0.5	0.979
Sr2	2b	1	0	0	0.5	0.2
Sr3	8g	1	0.5	0	0.151	0.2
Ni1	4e	1	0	0	0.251	0.2
O1	4c	0.69	0.5	0	0	1.8
O2	4e	0.94	0	0	0.134	1.8
O3	4e	1	0	0	0.628	1.8
O4	8f	1	0.25	0.25	0.25	1.8

$Sr_{2.5}Bi_{0.5}NiO_{4.79}$: [RS:O/D]-1201 (*P*4/*mmm*): *a* = 5.3711 Å, *c* = 17.586 Å

	Site	*g*	*x*	*y*	*z*	*B*
Bi1	1a	0.5	0	0	0	0.979
Sr1	1a	0.5	0	0	0	0.2
Bi2	1c	0.5	0.5	0.5	0	0.979
Sr2	1c	0.5	0.5	0.5	0	0.2
Bi3	1b	0	0	0	0.5	0.979
Sr3	1b	1	0	0	0.5	0.2
Bi4	1d	1	0.5	0.5	0.5	0.979
Sr4	1d	0	0.5	0.5	0.5	0.2
Sr5	4i	1	0.5	0	0.152	0.2
Sr6	4i	1	0.5	0	0.348	0.2
Ni1	2g	1	0	0	0.250	0.2
Ni2	2h	1	0.5	0.5	0.250	0.2
O1	2f	1	0	0.5	0	1.8
O2	2e	1	0	0.5	0.5	1.8
O3	2g	0.58	0	0	0.135	1.8
O4	2h	1	0.5	0.5	0.133	1.8
O5	2g	1	0	0	0.371	1.8
O6	2h	1	0.5	0.5	0.362	1.8
O7	8r	1	0.25	0.25	0.25	1.8

d d-perovskite in Figure S10d

$Sr_2BiNiO_{4.50}$: d-perovskite (*Pm*-3*m*): *a* = 8.3272 Å

	site	*g*	*x*	*y*	*z*	*B*
Sr1	8g	1	0.231	0.231	0.231	3.990
Ni1	1a	1	0	0	0	2.748
Ni2	3c	1	0	0.5	0.5	2.748
Bi1	3d	1	0.5	0	0	1.365
Bi2	1b	1	0.5	0.5	0.5	1.365
O1	6e	1	0.271	0	0	6.424
O2	12h	1	0.284	0.5	0	6.424

e O-1201 in Figure S10e

$Sr_{2.5}Bi_{0.5}NiO_{4.63}$: [RS:O/O]-1201 (*I*4/*mmm*): *a* = 5.3765 Å, *c* = 17.535 Å

	Site	*g*	*x*	*y*	*z*	*B*
Bi1	2a	1	0	0	0	0.979
Sr1	2a	0	0	0	0	0.2
Bi2	2b	0	0	0	0.5	0.979
Sr2	2b	1	0	0	0.5	0.2
Sr3	8g	1	0.5	0	0.151	0.2
Ni1	4e	1	0	0	0.251	0.2
O1	4c	0.69	0.5	0	0	1.8
O2	4e	0.94	0	0	0.134	1.8
O3	4e	1	0	0	0.628	1.8
O4	8f	1	0.25	0.25	0.25	1.8

$Sr_{2.5}Bi_{0.5}NiO_{4.79}$: [RS:O/D]-1201 (*P*4/*mmm*): *a* = 5.3700 Å, *c* = 17.605 Å

	Site	*g*	*x*	*y*	*z*	*B*
Bi1	1a	0.5	0	0	0	0.979
Sr1	1a	0.5	0	0	0	0.2
Bi2	1c	0.5	0.5	0.5	0	0.979
Sr2	1c	0.5	0.5	0.5	0	0.2
Bi3	1b	0	0	0	0.5	0.979
Sr3	1b	1	0	0	0.5	0.2
Bi4	1d	1	0.5	0.5	0.5	0.979
Sr4	1d	0	0.5	0.5	0.5	0.2
Sr5	4i	1	0.5	0	0.152	0.2
Sr6	4i	1	0.5	0	0.348	0.2
Ni1	2g	1	0	0	0.250	0.2
Ni2	2h	1	0.5	0.5	0.250	0.2
O1	2f	1	0	0.5	0	1.8
O2	2e	1	0	0.5	0.5	1.8
O3	2g	0.58	0	0	0.135	1.8
O4	2h	1	0.5	0.5	0.133	1.8
O5	2g	1	0	0	0.371	1.8
O6	2h	1	0.5	0.5	0.362	1.8
O7	8r	1	0.25	0.25	0.25	1.8

Table S10. Electronic heat capacity coefficients and Debye temperatures for O- and D-1201, estimated from Figure 4c.

Sample	γ (mJ/mol·K^2)	Θ_D (K)
O-1201	22.8(5)	250(24)
D-1201	23.9(6)	248(26)
